# An essential role of the autophagy activating kinase ULK1 in snRNP biogenesis

**DOI:** 10.1093/nar/gkab452

**Published:** 2021-06-07

**Authors:** Katharina Schmitz, Jan Cox, Lea Marie Esser, Martin Voss, Katja Sander, Antje Löffler, Frank Hillebrand, Steffen Erkelenz, Heiner Schaal, Thilo Kähne, Stefan Klinker, Tao Zhang, Luitgard Nagel-Steger, Dieter Willbold, Sabine Seggewiß, David Schlütermann, Björn Stork, Matthias Grimmler, Sebastian Wesselborg, Christoph Peter

**Affiliations:** Institute of Molecular Medicine I, Medical Faculty, Heinrich Heine University Düsseldorf, Düsseldorf, Germany; Institute of Molecular Medicine I, Medical Faculty, Heinrich Heine University Düsseldorf, Düsseldorf, Germany; Institute of Molecular Medicine I, Medical Faculty, Heinrich Heine University Düsseldorf, Düsseldorf, Germany; Institute of Molecular Medicine I, Medical Faculty, Heinrich Heine University Düsseldorf, Düsseldorf, Germany; Institute of Biochemistry, University of Cologne, Cologne, Germany; Institute of Molecular Medicine I, Medical Faculty, Heinrich Heine University Düsseldorf, Düsseldorf, Germany; Institute of Molecular Medicine I, Medical Faculty, Heinrich Heine University Düsseldorf, Düsseldorf, Germany; Institute of Virology, University Hospital Düsseldorf, Düsseldorf, Germany; Institute of Virology, University Hospital Düsseldorf, Düsseldorf, Germany; Institute for Genetics and Cologne Excellence Cluster on Cellular Stress Responses in Aging-Associated Diseases (CECAD), University of Cologne, Cologne, Germany; Institute of Virology, University Hospital Düsseldorf, Düsseldorf, Germany; Insitute of Experimental Internal Medicine, Otto von Guericke University, Magdeburg, Germany; Institut für Physikalische Biologie, Heinrich-Heine-Universität Düsseldorf, Germany; Institut für Physikalische Biologie, Heinrich-Heine-Universität Düsseldorf, Germany; Institute of Biological Information Processing (Structural Biochemistry: IBI-7), Forschungszentrum Jülich, Jülich, Germany; Institut für Physikalische Biologie, Heinrich-Heine-Universität Düsseldorf, Germany; Institute of Biological Information Processing (Structural Biochemistry: IBI-7), Forschungszentrum Jülich, Jülich, Germany; Institut für Physikalische Biologie, Heinrich-Heine-Universität Düsseldorf, Germany; Institute of Biological Information Processing (Structural Biochemistry: IBI-7), Forschungszentrum Jülich, Jülich, Germany; Institute of Molecular Medicine I, Medical Faculty, Heinrich Heine University Düsseldorf, Düsseldorf, Germany; Institute of Molecular Medicine I, Medical Faculty, Heinrich Heine University Düsseldorf, Düsseldorf, Germany; Institute of Molecular Medicine I, Medical Faculty, Heinrich Heine University Düsseldorf, Düsseldorf, Germany; Hochschule Fresenius, Idstein, Germany; DiaSys Diagnostic Systems GmbH, Alte Strasse 9, 65558 Holzheim, Germany; Institute of Molecular Medicine I, Medical Faculty, Heinrich Heine University Düsseldorf, Düsseldorf, Germany; Institute of Molecular Medicine I, Medical Faculty, Heinrich Heine University Düsseldorf, Düsseldorf, Germany

## Abstract

The biogenesis of small uridine-rich nuclear ribonucleoproteins (UsnRNPs) depends on the methylation of Sm proteins catalyzed by the methylosome and the subsequent action of the SMN complex, which assembles the heptameric Sm protein ring onto small nuclear RNAs (snRNAs). In this sophisticated process, the methylosome subunit pICln (chloride conductance regulatory protein) is attributed to an exceptional key position as an ‘assembly chaperone’ by building up a stable precursor Sm protein ring structure. Here, we show that—apart from its autophagic role—the Ser/Thr kinase ULK1 (Uncoordinated [unc-51] Like Kinase 1) functions as a novel key regulator in UsnRNP biogenesis by phosphorylation of the C-terminus of pICln. As a consequence, phosphorylated pICln is no longer capable to hold up the precursor Sm ring structure. Consequently, inhibition of ULK1 results in a reduction of efficient UsnRNP core assembly. Thus ULK1, depending on its complex formation, exerts different functions in autophagy or snRNP biosynthesis.

## INTRODUCTION

Splicing of mRNA precursors is essential for the maintenance and function of the cellular proteome. This ubiquitous process is mediated by RNA–protein complexes termed spliceosomal U-rich small nuclear ribonucleoprotein particles (UsnRNPs), which are composed of one specific small nuclear RNA (snRNA) and a heptameric ring of the seven common (Sm) proteins B, D1, D2, D3, E, F and G (1–3). The assembly of the UsnRNPs is a sophisticated and stepwise process regulated by the protein arginine methyltransferase 5 (PRMT5), also known as methylosome, and the survival motor neuron (SMN) complex (4–11).

In this segmented process, the methylosome subunit pICln is attributed to an exceptional key position as an ‘assembly chaperone’ (1,12,13). During this assembly reaction pICln functions as a kinetic trap by building up a stable heterohexameric precursor ring structure together with the Sm proteins D1, D2, E, F and G (13). For the consecutive assembly reaction of the UsnRNP core, it is essential that catalytic snRNA is transferred onto this ring structure with the help of the SMN complex. *In vitro* as well as by *in vivo* experimental systems / cellular extracts the completion of UsnRNP assembly strongly depends on metabolic energy by ATP hydrolysis (8,14). However, the involved regulating elements and the detailed structural and molecular mechanism of ATP-dependent UsnRNP core assembly have remained elusive to date.

Here, we identify the autophagy activating Ser/Thr Unc-51-like kinase (ULK1) as a novel key regulator in this process. We demonstrate that pICln is a specific new substrate of ULK1 and that the newly identified phosphorylation sites in the C-terminus of pICln are responsible for breaking up the Sm ring structure at the newly identified SmG-pICln contact surface. We demonstrate that phosphorylation of pICln by ULK1 is an essential regulatory step to promote efficient biogenesis of the UsnRNP. Thus, we show that ULK1 comprises a crucial key function in two distinct cellular processes: autophagy as well as UsnRNP biogenesis, a process which is known to be highly dependent on protein methylation and phosphorylation events (7,15,16).

## MATERIALS AND METHODS

### Antibodies

The following primary antibodies were used for immunoblotting and immunofluorescence: α-Actin (A5316, Sigma Aldrich), α-ATG3 (3415, CST), α-ATG101 (SAB4200175, Sigma-Aldrich), α-ATG13 (M183-3, MBL; SAB4200100, Sigma-Aldrich), α-ATG14 (PD026, MBL), α-ATG14 pS29 (92340, CST), α-Coilin (PA5-29531, Invitrogen), α-FIP200 (A301-574A, Bethyl), α-GAPDH (ab8245, Abcam), α-GFP (3H9, Chromotek), α-LC3B (2775, CST), α-p62 (GP62-C, PROGEN), α-pICln (sc-393525, Santa Cruz), α-PRMT5 (2252, CST), α-SmB (S0698, Sigma-Aldrich), α-SmD1 (ab79977, Abcam), α-SmD2 (SAB2102257, Sigma-Aldrich), α-SmE (NBP2-43792, Novus), α-SmF (SAB2102258, Sigma-Aldrich), α-SmG (HPA064152, Sigma-Aldrich), α-SMN (clone 2B1, 05-1532, Merck Millipore), α-SNRPB (Y12, MA5-13449, Invitrogen), α-Tubulin (clone B512, T5168, Sigma Aldrich), α-ULK1 (8054; CST), α-ULK2 (ab97695, Abcam), α-WD45 (2823, CST). The detection of proteins was carried out with the following fluorescent secondary antibodies: IRDye 680LT goat α-rabbit, IRDye 680LT goat α-mouse, IRDye 800CW donkey α-rabbit, IRDye 800CW donkey α-mouse, IRDye 800CW goat α-rat. For the detection of proteins *in vivo* via IF the following secondary antibodies were used: Alexa Fluor 568 donkey anti-mouse (A10037, Invitrogen) and Alexa Fluor 647 donkey anti-rabbit (A31573, Invitrogen).

### Plasmids and proteins

For *in vitro* assays and pulldown experiments plasmids encoding full-length cDNAs of pICln (X91788.1), SmB (X17567.1), SmD3 (U15009.1) and SmG (X85373.1) were cloned from HEK293T, HeLa, or Jurkat cDNA (High Capacity cDNA Reverse Transcription Kit, Applied Biosystems) into pET-28a (69864-3, EMD Biosciences) or pGEX-6P-1 (27-4597-01, Amersham) with the following primers: pICln, 5′-GGATCCATGAGCTTCCTCAAAAGTTTCCC-3′ and 5′-GTCTCGAGTCAGTGATCAACATCTGCATCC-3′; SmB, 5′-ATGAATTCATGACGGTGGGCAAGAGC-3′ and 5′ATGCGGCCGCTCAAAGAAGGCCTCGCATC-3′; SmD3, 5′ATGAATTCATGTCTATTGGTGTGCCG-3′ and 5′-ATCTCGAGTTATCTTCGCTTTTGAAAGATG-3′; SmG, 5′-ATGGAATTCATGAGCAAAGCTCACCCT-3′ and 5′ATGCTCGAGTTATCGTTCCAAGGCTT-3′. For cloning the pICln phosphorylation mutants the Pfu DNA Polymerase (Promega) and the following primers were used: S193, 195, 197A, 5′-GATTAGAAGGAATGCTTGCTCAGGCTGTGGCCAGCCAGTATAATATG-3′ and 5′-CATATTATACTGGCTGGCCACAGCCTGAGCAAGCATTCCTTCTAATC-3′; S193, 195, 197D, 5′-GATTAGAAGGAATGCTTGATCAGGATGTGGACAGCCAGTATAATATG-3′ and 5′-CATATTATACTGGCTGTCCACATCCTGATCAAGCATTCCTTCTAATC-3′; S193A, 5′-ATGCTTGCTCAGTCTGTGAGCAGCCAGTATAATATGGCTG-3′ and 5′-CAGACTGAGCAAGCATTCCTTCTAATCTCTCCAGTGTGG-3′; S195A, 5′-TTCTCAGGCTGTGAGCAGCCAGTATAATATGGCTGGGGTC-3′ and 5′-GCTCACAGCCTGAGAAAGCATTCCTTCTAATCTCTCCAG-3′; S197A, 5′-CAGTCTGTGGCCAGCCAGTATAATATGGCTGGGGTCAGG-3′ and 5′-GGCTGGCCACAGACTGAGAAAGCATTCCTTCTAATCTC-3′; S193D, 5′-ATGCTTGATCAGTCTGTGAGCAGCCAGTATAATATGGCTG-3′ and 5′-CAGACTGATCAAGCATTCCTTCTAATCTCTCCAGTGTGG-3′; S195D, 5′-TTCTCAGGATGTGAGCAGCCAGTATAATATGGCTGGGGTC-3′ and 5′-GCTCACATCCTGAGAAAGCATTCCTTCTAATCTCTCCAG-3′; S197D 5′-CAGTCTGTGGACAGCCAGTATAATATGGCTGGGGTCAGG-3′ and 5′-GGCTGTCCACAGACTGAGAAAGCATTCCTTCTAATCTC-3′;.

Generation of pGEX6P-1-PRMT5; -WD45 (17); -SmD1; pET28a-SmD1 (18); pcDNA-FRT-TO-GFP; -GFP-ULK1; -GFP-ULK1kd; -GFP-ULK1/ΔCTD (19) and pMSCVbsd/GFP-ULK1 plasmids have been described previously (20). pET100/D-TOPO-SmE; -SmF; -SmD2 and pcDNA5-FRT-TO-GFP-ULK1 C-terminal domain (AA828-1050) and ULK1-GABARAP domain (AA287–416) were synthesized by GeneArt (Thermo Fisher Scientific).

For interaction studies, pMSCVbsd/GFP and pMSCVbsd/GFP-ULK1 kinase domain plasmids were generated by using pMSCVbsd/GFP-ULK1 and the following primers: 5′-CATGGACGAGCTGTACAAGTGAGGACTCGGATCCCTGGAG-3′ (GFP) and 5′- GTTTTTTCATCACCCTTTCTAACTCGATGCCAGCCCC-3′ (ULK1 kinase domain).

For purification of GST-tagged proteins and pulldown assays glutathione sepharose 4B from GE Healthcare was used. For HIS-tagged proteins HisPur™ Ni-NTA Resin from Thermo Fischer was used. For pulldown assays, recombinant proteins were pre-incubated 1.5 h at 4°C. Subsequently, sepharose was added and further incubated for one hour. After washing, analysis of the respective immobilized proteins per SDS-PAGE and western blotting with specific antibodies was performed.

Recombinant active ULK1, ULK2 and PRMT5 were purchased from Sigma-Aldrich (SRP5096, SRP5097, SRP0145). ULK1/2 inhibitor MRT67307 was obtained through the MRC PPU Reagents and Services facility (MRC PPU, College of Life Sciences, University of Dundee, Scotland, mrcppureagents.dundee.ac.uk).

### Cell lines and cell culture

Generation of inducible Flp-In T-REx 293 cells system expressing GFP, GFP-ULK1, GFP-ULK1kd, GFP-ULK1/ΔCTD and GFP-pICln were carried out according to the manufacturer's instructions (Invitrogen, Thermo Fisher Scientific) and has been described previously (19). For induction of GFP, GFP-ULK1, GFP-ULK1kd and GFP-ULK1/ΔCTD expression, Flp-In T-REx 293 cell lines were stimulated with 0.1 μg/ml Doxycycline (Clontech) for 18 h. For starvation treatment, cells were incubated in Earle's Balanced Salt Solution (EBSS; Gibco, Thermo Fisher Scientific) for 1 h. For ULK1 knockdown HEK293T cells were transfected using DharmaFECT1 (GE Dharmacon) with 50 nM ULK1 siRNA (L-005049-00-0010, SMARTpool, ON-TARGETplus, GE Dharmacon), 50 nM ULK2 siRNA (L-005396-00-0005, SMARTpool, ON-TARGETplus), and 50 nM of the control siRNA (D-0018-10-1020, SMARTpool, On target plus non-targeting pool) for 48 h.

For interaction studies, HEK293T was transiently transfected with pcDNA5-FRT-TO-GFP-ULK1 GABARAP domain and -ULK1 C-terminal domain constructs using Lipofectamine 3000 (Invitrogen, L3000-015). The cells were harvested 24 h after transfection. Additionally, HEK293T cells stably expressing the GFP-ULK1 kinase domain and GFP were generated. Therefore Plat-E cells were used as packaging cell line and transfected with the retroviral pMSCVbsd expression vectors using FuGENE6 (Promega, E2692). HEK293T cells were incubated with the retroviral supernatant containing 3 mg/ml Polybrene (Sigma-Aldrich, H9268-106) and selected with Blasticidin.

All cell lines were cultured in DMEM (4.5 g/l d-glucose; Gibco, Thermo Fisher Scientific) supplemented with 10% (v/v) FCS (Biochrom, Merck), 100 U/ml Penicillin, and 100 μg/ml Streptomycin (Gibco, Thermo Fisher Scientific) in a 5% CO_2_ humidified atmosphere at 37°C. For the retroviral transduction Mouse Embryonic Fibroblast (MEF) cells lacking ULK1/2 first Plat E cells were transfected with pMSCV- based expression vectors with FuGENE6 to reconstitute the cells with ULK1 (human) or ULK2 (mouse) for 48 h at 37°C and 5% CO_2_. In a second step, the retroviral supernatant was added to the MEF ULK1/2 DKO cells for reconstitution for 72 h and afterward selected in DMEM high glucose media with 5 μg/ml Puromycin.

### Protein expression and purification

Proteins were overexpressed in BL21 competent *E. coli* for 4 h at RT after induction with 1 mM IPTG. Cells were lysed in 300 mM NaCl, 50 mM Tris/HCl pH 7.5, 5 mM EDTA, 5 mM EGTA, 0.01% (v/v) Igepal, protease inhibitors (cOmplete, EDTA-free protease inhibitor cocktail tablets, Roche), 50 mg/ml Lysozyme (Serva) and by sonication. After centrifugation at 10 000 g for 30 min the lysate was incubated with glutathione sepharose 4B (GE Healthcare) for 1.5 h at 4°C and subsequently washed 3 times with lysis buffer.

### Immunoblotting and immunopurification

Protein amounts of cleared S100 cytoplasm extract were determined by the Bradford method. Samples were separated by Tris/Tricine or Tris/Glycine SDS gel electrophoresis (21) and transferred to PVDF membranes (Immobilon-FL, Merck Millipore). The immunoblot analysis was performed using the indicated antibodies and signals were detected with an Odyssey LI-COR Imaging System. For GFP immunopurification S100 extracts were incubated with GFP-Trap_A beads (ChromoTek) at 4°C for at least 1.5 h with rotation. Purified proteins were washed 3 times with washing buffer (lysis buffer without Triton X-100 and protease inhibitors), eluted in sample buffer [375 mM Tris pH 7.5; 25.8% (w/v) glycerol; 12.3% (w/v) SDS; 0.06% (w/v) Bromophenol blue; 6% (v/v) β-mercaptoethanol; pH 6.8] and analyzed by immunoblotting. For endogenous immunopurification protein-G-sepharose (GE Healthcare) was washed 3× with HBSS and in following coated with 1 μg ULK1 antibody. Size exclusion fractions were incubated with the coated sepharose at 4°C overnight with rotation. Coated sepharose was washed three times with washing buffer (see above) and analyzed by immunoblotting. To generate cleared cellular lysates, cells were lysed in lysis buffer (50 mM Tris-HCl, pH 7.5, 150 mM NaCl, 1 mM EDTA, 1 mM Na_3_VO_4_, 50 mM NaF, 5 mM Na_4_P_2_O_7_, 1% [v/v] Triton X-100, and protease inhibitor cocktail (Sigma-Aldrich, #P2714) for 30 minutes on ice. Lysates were cleared by centrifugation at 18 000 rpm and 4°C for 15 min.

### Immunofluorescence microscopy

HEK293T and Flp-In T-REx 293 cells were seeded on coverslips in DMEM high glucose media (4.5 g/l d-glucose) with 10% (v/v) FCS (Biochrom, Merck), 100 U/ml Penicillin, and 100 μg/ml Streptomycin (Gibco, Thermo Fisher Scientific) one day before staining. On the next day after washing the cells once with Dulbecco's phosphate-buffered saline (DPBS), fixing was performed with 4% paraformaldehyde for 10 min. Cells were permeabilized with 0.2% Triton X-100/PBS for 10 min and blocked with 5% BSA for 30 minutes. Proteins were detected with anti-SMN clone 2B1 (1:1000) and anti-Coilin antibody (1:1000), incubation time 2 h. As a secondary antibody, Alexa Fluor 568 (1:200; shown in green) and Alexa Fluor 647 (1:200; shown in red) were used. Analysis of the staining was performed with the ZEISS Apotome.2 and a 40× oil immersion objective. For each counted cell a z-stack with every five pictures per cell, one stack every 0.2 μm, was analyzed.

### Cytoplasm extraction (S100) and size exclusion chromatography

HEK293T cells were incubated with Roeder A buffer (22) in an appropriate amount for 10 min at room temperature, dounced 10 times, and adjusted to 150 mM NaCl. After centrifugation at 17 000 g for 30 min the supernatants (S100 extracts) were filtrated with Millex-HA, 0.45 μm filter unit (Merck Millipore) and applied to a Superdex 200 HiLoad 16/600 or Superdex 200 increase 10/300 GL column (GE Healthcare). 2 ml respectively 0.5 ml fractions were collected in running buffer (150 mM NaCl, 50 mM Tris/HCl pH 7.5) and analyzed by immunoblotting. The columns were calibrated with thyroglobulin (669 kDa), ferritin (440 kDa), catalase (232 kDa), aldolase (158 kDa), albumin (67 kDa), ovalbumin (43 kDa), and RNase (14 kDa) (GE Healthcare).

### *In vitro* phosphorylation

GST-PRMT5, -WD45 and -pICln were purified from *E. coli*, GFP-ULK1, and GFP-ULK1kd from Flp-In T-REx 293 cells. GST-ULK1 (1–649) and GST-ULK2 (1-631) were used from Sigma-Aldrich. Recombinant active GST-ULK1/2 or purified GFP-ULK1 and GST substrate in appropriate amounts were incubated in 2 μM ATP, 10 μCi [32P]-ATP (Hartmann Analytic), 2.5 mM Tris/HCl pH 7.5, 5 μM EGTA, 50 μM DTT and 3.75 mM Mg(CH_3_COO)_2_ for 45 min at 30°C. Gel filtration fractions were incubated 1:1 with kinase buffer (50 mM NaCl, 25 mM Tris/HCl pH 7.5, 10 mM MgCl_2_, 2 mM CaCl_2_), GST-pICln and 10 μCi [32P]-ATP for 45 min at 30°C and washed three times with kinase washing buffer (300 mM NaCl, 50 mM Tris/HCl pH 7.5, 5 mM EGTA, 5 mM EDTA, 0.01% (v/v) Igepal). The reaction was terminated by adding sample buffer, samples were subjected to SDS-PAGE, and after coomassie staining autoradiography was performed.

### *In vitro* translation and interaction assay

[35S]methionine-labelled (Hartmann Analytic) proteins were made using the TNT Quick Coupled Transcription/Translation System (Promega). For binding assay *in vitro* translated proteins were incubated with GST fusion proteins bounded on glutathione sepharose 4B (GE Healthcare) in interaction buffer (300 mM NaCl, 50 mM Tris/HCl pH 7.5, 1 mM EGTA, 1 mM EDTA, 1 mM DTT and 0.01% (v/v) Igepal) for 1.5 h at 4°C under rotation. After washing 2 times with interaction buffer bounded proteins were eluted by adding sample buffer, separated by SDS-PAGE, and analyzed by coomassie staining and autoradiography.

### *In vitro* methylation

Target proteins were purified from *E. coli*, GFP-ULK1 from Flp-In T-REx 293 cells. Active PRMT5 (Active Motif/Sigma-Aldrich) and an appropriate amount of target proteins were incubated in 1 μCi adenosyl-l-methionine, *S*-[methyl-3H] (Perkin Elmer/Hartmann-Analytic), 50 mM Tris/HCl pH 7.5, 1 mM EGTA and 1 mM EDTA for 1–1.5 h at 37°C. The reaction was terminated by adding sample buffer, samples were subjected to SDS-PAGE. After coomassie staining or western blotting and subsequent amido black staining, autoradiography was performed.

### *In vitro* transcription and assembly of UsnRNPs

The U1 snRNA was *in vitro* transcribed and labelled with 10 μCi [32P]-UTP (Hartmann Analytic). For the analysis of the UsnRNP assembly *in vitro*, a GFP-pICln immunoprecipitation was performed with 1 mg cytoplasmic extract (S100). The efficiency of the UsnRNP biogenesis of this immunoprecipitation was analyzed by adding U1 snRNA to the immunoprecipitation in the presence/absence of ATP and active ULK1, or ULK2 kinase. The assembly of UsnRNPs in S100 extracts was performed with 50 μg of S100 extract. The reactions were incubated at 35°C for 45 min with 800 counts [32P]-UTP labeled, U1 snRNA, 2 μg t-RNA, 5 mM ATP and 1 μl RNAsin in a final volume of 20 μl PBS. The assembly reactions were analyzed by a native RNA gel electrophoresis and subsequent autoradiography.

### Analysis of ULK1 mediated pICln phosphorylation by LC-MS/MS

Samples were separated by SDS-PAGE after *in vitro* kinase assay. Gel areas containing GST-pICln were excised and subjected to in-gel digestion in an adapted manner according (23). NanoLC-MS/MS analysis was performed on a hybrid dual-pressure linear ion trap/orbitrap mass spectrometer (LTQ Orbitrap Velos Pro, Thermo Fisher Scientific) equipped with a U3000 nano-flow HPLC (Thermo Fisher Scientific) as described (24). The procedure in brief: Samples were separated on a 75 μm I.D., 25 cm PepMap C18-column (Dionex Thermo Fisher Scientific) applying a gradient from 2% (v/v) ACN to 35% (v/v) ACN in 0.1% (v/v) formic acid over 95 min at 300 nl/min. The LTQ Orbitrap Velos Pro MS used exclusively CID-fragmentation with wideband activation (pseudo-MS3 for neutral losses of phosphate residues) when acquiring MS/MS spectra. The spectra acquisition consisted of an orbitrap full MS scan (FTMS; resolution 60 000; *m*/*z* range 400–2000) followed by up to 15 LTQ MS/MS experiments (Linear Trap; minimum signal threshold: 500; wideband isolation; dynamic exclusion time setting: 30 s; singly-charged ions were excluded from selection, normalized collision energy: 35%; activation time: 10 ms). Raw data processing, protein identification, and phosphopeptide assignment of the high-resolution orbitrap data were performed by PEAKS Studio 7.0 (Bioinformatics Solutions Inc.). The false discovery rate (FDR) was set to <1%. Phosphorylation sites were accepted as confident for *P* < 0.005 (modified *t*-test, included in PEAKS Studio 7.0) and PhosphoRS score > 90 (25).

### Analysis of pICln complex formation by quantitative LC-MS/MS

5 μg of GST-pICln wt and mutants were incubated with 1 mg HEK293T S100 extract overnight at 4°C. After the addition of 30 μl glutathione sepharose 4B (GE Healthcare) and further incubation at 4°C for 4 h, purified proteins were washed three times with washing buffer (see above) and three times with PBS. Then GST-pICln bound to GSH-beads were subjected to ‘on beads digestion’ as described earlier (26). The procedure in brief: GST-pICln bound to beads were resuspended in 50 mM ammonium bicarbonate. Cysteines were reduced by adding 2 mM dithiothreitol (DTT) for 30 min at room temperature and subsequently β-methylthiolated by addition of 10 mM methylmethanethiosulfonate (MMTS). Digestion was performed by the addition of 0.5 μg trypsin (Promega) and incubation overnight at 37°C. Peptides were extracted by pooling the primary supernatant and the supernatant of a subsequent washing step using 0.1% (v/v) trifluoroacetic acid (TFA). Peptides were purified with reversed-phase C18 ZipTip nano-columns (EMD Millipore), eluted with 0.1% (v/v) TFA/70% (v/v) ACN, and dried. Protein identification was performed by high-resolution mass spectrometry on a hybrid dual-pressure linear ion trap/orbitrap mass spectrometer as described above. Relative protein quantification was achieved using Skyline analysis platform (27) for MS-peak integration on extracted ion chromatograms of the following selected peptides: sp|P54105|ICLN_HUMAN: K.GLGTGTLYIAESR.L [30, 42], K.FEEESKEPVADEEEEDSDDDVEPITEFR.F [85, 112], R.LEGMLSQSVSSQYNMAGVR.T [187, 205]; sp|P62308|RUXG_HUMAN: R.GNSIIMLEALER.V [63, 74], R.GNSIIMLEALER.V [63, 74]; sp|P62306|RUXF_HUMAN: R.CNNVLYIR.G [65, 72], sp|P62304|RUXE_HUMAN: K.VMVQPINLIFR.Y [12, 22], K.GDNITLLQSVSN.- [80, 91]; sp|P62314|SMD1_HUMAN: K.LSHETVTIELK.N [9, 19], K.NREPVQLETLSIR.G [48, 60], R.YFILPDSLPLDTLLVDVEPK.V [66, 85]; sp|P62316|SMD2_HUMAN: K.NNTQVLINCR.N [37, 46], R.GDSVIVVLR.N [102, 110]; sp|P62318|SMD3_HUMAN: R.VAQLEQVYIR.G [54, 63], R.FLILPDMLK.N [69, 77]; sp|P14678|RSMB_HUMAN: R.VLGLVLLR.G [65, 72], R.GENLVSMTVEGPPPK.D [73, 87]. pICln peptides have been used for internal normalization.

### Surface plasmon resonance

The affinity dissociation constants (*K*_d_) of pICln wt and pICln aspartate mutant for SmG were determined by surface plasmon resonance (SPR) using a T200 device (Biacore, GE Healthcare). The immobilization of the ligand was performed under mild acidic condition by dissolving the respective protein stock solution (50 mM HEPES, 150 mM NaCl, 1 mM EDTA, pH 7.0) in 10 mM sodium acetate buffer pH 5.0 and injection onto the N-hydroxysuccinimide (NHS)/1-ethyl-3-(3 dimethylaminopropyl)carbodiimide (EDC) activated series S CM5 sensor chip (Biacore, GE Healthcare) surface. By sequential injection of several μl of ligand solvent, the target immobilization level of 400 RU for the 51 kDa ligand GST-pICln was reached. The remaining activated surface of the ligand flow cell, as well as the activated reference flow cell, was blocked by the injection of 1 M ethanolamine pH 8.5 for 7 min. Affinity measurements were performed in running buffer (300 mM NaCl, 50 mM Tris/HCl pH 7.5, 1 mM EDTA and 1 mM EGTA). The analytes were stored in purification buffer and freshly dissolved in sample buffer (300 mM NaCl, 50mM Tris/HCl pH7.5, 1 mM EDTA, 1 mM EGTA, 1 mg/ml BSA, 1 mM DTT and 0.01% (v/v) Igepal) before analysis. During measurement analyte samples were sealed against evaporation and stored at 10°C until injection. For affinity determination of the analytes, multi-cycle kinetic experiments were applied at 20°C and 10 μl min−1 flow rate. In between each cycle, a regeneration command (200 s injection of 10 mM glycine pH 10 at 30 μl min^–1^, followed by a stabilization period of 500 s with running buffer) was executed if dissociation phase time was not sufficient to dissociate the formed complex of ligand and analyte. Association and dissociation phase of 100 s and 600 s for GST-pICln—SmG analysis was chosen. As a quality control for the activity of the used analyte batch, wild type, and respective mutants were run sequentially on the same sensor chip. The reference flow cell and buffer cycles were used for double referencing of the sensorgrams. For evaluation, the sensorgrams were fitted applying the steady-state fit model of the Biacore T200 Evaluation Software 2.0 (GE Healthcare) and the offset was constantly set to zero.

### Sedimentation velocity analysis

Sedimentation velocity analytical ultracentrifugation (SV-AUC) was carried out using a ProteomLab XL-A ultracentrifuge (Beckman Coulter, Brea, CA, USA) equipped with a fluorescence detection system (Aviv Biomedical inc., Lakewood, NJ, USA). Samples were filled into 3-mm double-sector titanium cells (Nanolytics, Potsdam, Germany) with a volume of 100 μl, respectively. Quartz windows were used for all cell assemblies. Radial fluorescence scans were collected continuously at 40 000 rpm using a 488 nm laser for excitation and a 520 nm cut-off emission filter using a constant photomultiplier voltage. A radial resolution of 20 μm was used for data acquisition. The gains for all samples were adjusted for optimum signal-to-noise ratios. All samples were thermally equilibrated to 20°C for about 1.5 hours before starting the measurement. The experiments were performed at 40,000 rpm (equivalent to 129 024 × *g*) at 20°C for 5 h. All SV data was then analyzed with continuous distribution *c*(*s*) Lamm equation model with maximum entropy regularization, which is implemented in the software package SEDFIT (version 15.01b, http://www.analyticalultracentrifugation.com/) (28). The fitting parameters, including the partial specific volumes (${\rm{\bar v}}$), buffer density (ρ), and viscosity (η) were calculated based on the protein sequences and buffer composition, respectively, by applying SEDNTERP (version 20130813 BETA, http://bitcwiki.sr.unh.edu/index.php/Main_Page). The size distributions as well as the sedimentation profiles were presented by GUSSI (version 1.2.1) (29). The final sedimentation coefficients were normalized to the s-values at 20°C in pure water solvent (s20, w).

## RESULTS

### ULK1 is a new interaction partner of the PRMT5 complex

To identify new interaction partners of ULK1 we established an inducible expression system of GFP-ULK1 in Flp-In T-REx 293 cells. Utilizing this system, we identified and characterized new substrates of ULK1 by subsequent co-immunopurification and mass spectrometry like the AMP-activated protein kinase (α, β, and γ AMPK) (19). This survey also revealed a putative association of ULK1 with the Protein arginine N-methyltransferase 5 (PRMT5) as well as its binding partners the methylosome protein 50 (MEP50/WD45) and the chloride conductance regulatory protein (pICln).

**Figure 1.**
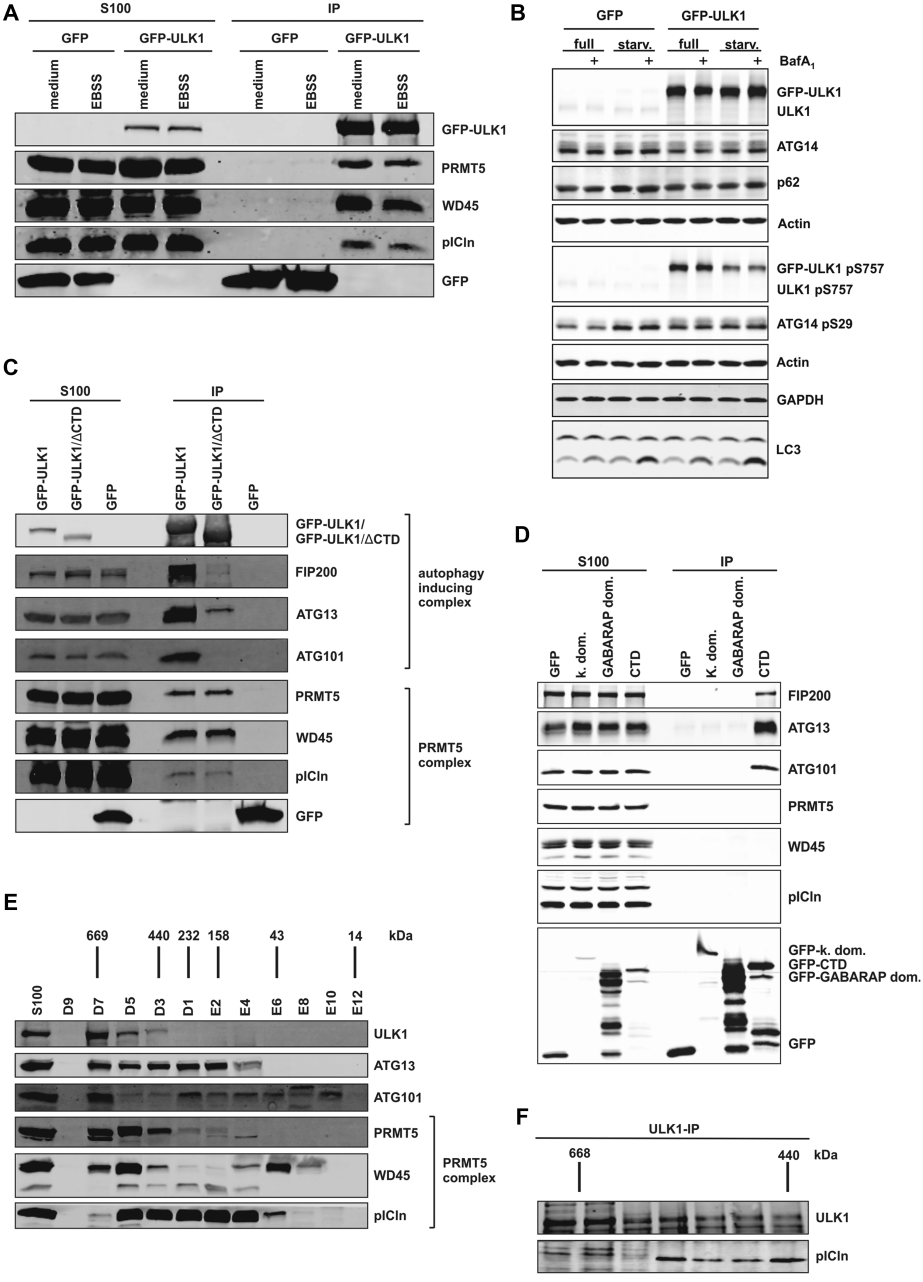
ULK1 interacts directly with the PRMT5 complex independent of its role in autophagy. (**A**) Flp-In T-REx 293-GFP-ULK1 and Flp-In T-REx 293-GFP cells were stimulated with 0.1 μg/ml doxycycline for 18 h, followed by 1 h of starvation treatment with EBSS. After cytoplasm extraction (S100) GFP-IP was performed and analyzed by Tris/Glycine-SDS-PAGE and western blotting using antibodies against GFP, PRMT5, WD45, and pICln. (**B**) Flp-In T-REx 293-GFP-ULK1 and Flp-In T-REx 293-GFP cells were stimulated with 0.1 μg/ml doxycycline for 18 h, followed by treatment with full or starvation medium (EBSS) in the absence or presence of bafilomycin A1 (BafA1; 10 nM) for 1 h. Afterward, cells were harvested, lysed, and cleared cellular lysates were subjected to Tris/Glycine-SDS-PAGE and immunoblotting for GFP, ULK1 pS757, ATG14, ATG14 pS29, p62, LC3, GAPDH, and Actin. (**C**) Flp-In T-REx 293-GFP-ULK1, -GFP-ULK1/ΔCTD and -GFP cells were stimulated with 0.1 μg/ml doxycycline for 18 h. After cytoplasm extraction (S100) GFP-IP was performed and analyzed by Tris/Glycine-SDS-PAGE and western blotting using antibodies against FIP200, GFP, PRMT5, ATG13, WD45, pICln and ATG101. (**D**) HEK293T cells were stably transfected with pMSCVbsd-GFP-ULK1 kinase domain (k. dom.) and pMSCVbsd-GFP constructs. Additionally, HEK293T cells were transiently transfected with pcDNA5-FRT-TO-GFP-ULK1 GABARAP domain (GABARAP dom.) and -ULK1 C-terminal domain (CTD) constructs. After cytoplasm extraction (S100) GFP-IP was performed and analyzed by Tris/Glycine-SDS-PAGE and western blotting using antibodies against GFP, FIP200, ATG13, ATG101, PRMT5, WD45, and pICln. (**E**) S100 extract was generated of HEK293T cells and applied to a Superdex 200 increase column. Fractions were analyzed by Tris/Glycine-SDS-PAGE and immunoblotting using antibodies against ULK1, ATG13, ATG101, PRMT5, WD45 and pICln. (**F**) S100 extract of HEK293T cells was applied to a Superdex 200 increase column and subsequent immunoprecipitation of endogenous was performed with antibody against ULK1. Immunoprecipitation was analyzed by Tris/Glycine-SDS-PAGE using antibodies against ULK1 and pICln.

To validate our mass spectrometry results we performed immunoblot analysis from Flp-In T-REx 293 cells expressing inducible GFP-ULK1 or a GFP control. Only immunopurification of GFP-ULK1 but not GFP alone revealed an interaction with PRMT5, WD45 and pICln (Figure 1A). Furthermore, the interaction of the methylosome with ULK1 seems to be independent of autophagy induction since PRMT5 and its binding partners were co-immunopurified to the same extent upon incubation with starvation medium for induction of autophagy (Figure 1A, B). During autophagy, ULK1 is associated with ATG13, ATG101 and RB1CC1/FIP200 in a high molecular weight complex of 2 MDa (30). The formation of this autophagy-inducing complex requires the C-terminal domain (CTD) of ULK1 (31). When we used Flp-In T-REx 293 cells inducibly expressing a mutant lacking the CTD of ULK1 (GFP-ULK1/ΔCTD) we still can co-immunopurify comparable amounts of PRMT5, WD45, and pICln (Figure 1C). In contrast ATG13, ATG101 and FIP200 could only be co-immunopurified with full-length ULK1 (Figure 1C) or with the C-terminal domain of ULK1 (Figure 1D). Thus, the interaction of ULK1 with the methylosome is independent of the C-terminal domain of ULK1 and additionally independent of its kinase and GABARAP domain (Figure 1D). In addition, recombinant co-purified ULK1 and pICln confirmed these results and showed that ULK1 directly interacts with pICln (SD Figure 1A). This intriguing observation suggests that apart from its central role in autophagy, ULK1 may also play a crucial part in UsnRNP biogenesis and activity.

Furthermore to the established and well-characterized autophagy-inducing complex with a size of >2000 kDa (30), overexpressed ULK1 can be detected in a smaller population in a CTD-independent manner with a molecular mass of 400–500 kDa (31). Since the PRMT5 complex also has a molecular size of 400–600 kDa (4,5), we used Superdex 200 increase column size exclusion chromatography to analyze the co-migration of ULK1 with the PRMT5 complex. Consistent with the results from Chan and coworkers (31) we detected endogenous ULK1 from HEK293T cells in a size range of 400–600 kDa (Figure 1E). We could identify the co-migration of ULK1 with the endogenous PRMT5 complex in these fractions (Figure 1E). Additionally, we also could co-immunopurify endogenous pICln along with endogenous ULK1 after size-exclusion chromatography in the corresponding fractions of ULK1 / pICln co-migration (Figure 1F).

These results posed the question of whether ULK1 is a new substrate of the methylosome. Subsequent radioactive *in vitro* methylation assays with active PRMT5 could not provide evidence for this, whereas the Sm protein D1, a known substrate of PRMT5 (5), was efficiently methylated (SD Figure 1B).

### The methylosome subunit pICln is a new substrate of the autophagy initiating kinase ULK1

Recent studies have shown that besides methylation UsnRNP biogenesis crucially depends on ATP levels (8,14–16). Moreover, the PRMT5 complex subunit pICln is highly phosphorylated *in vitro* and *in vivo* (15,32).

**Figure 2.**
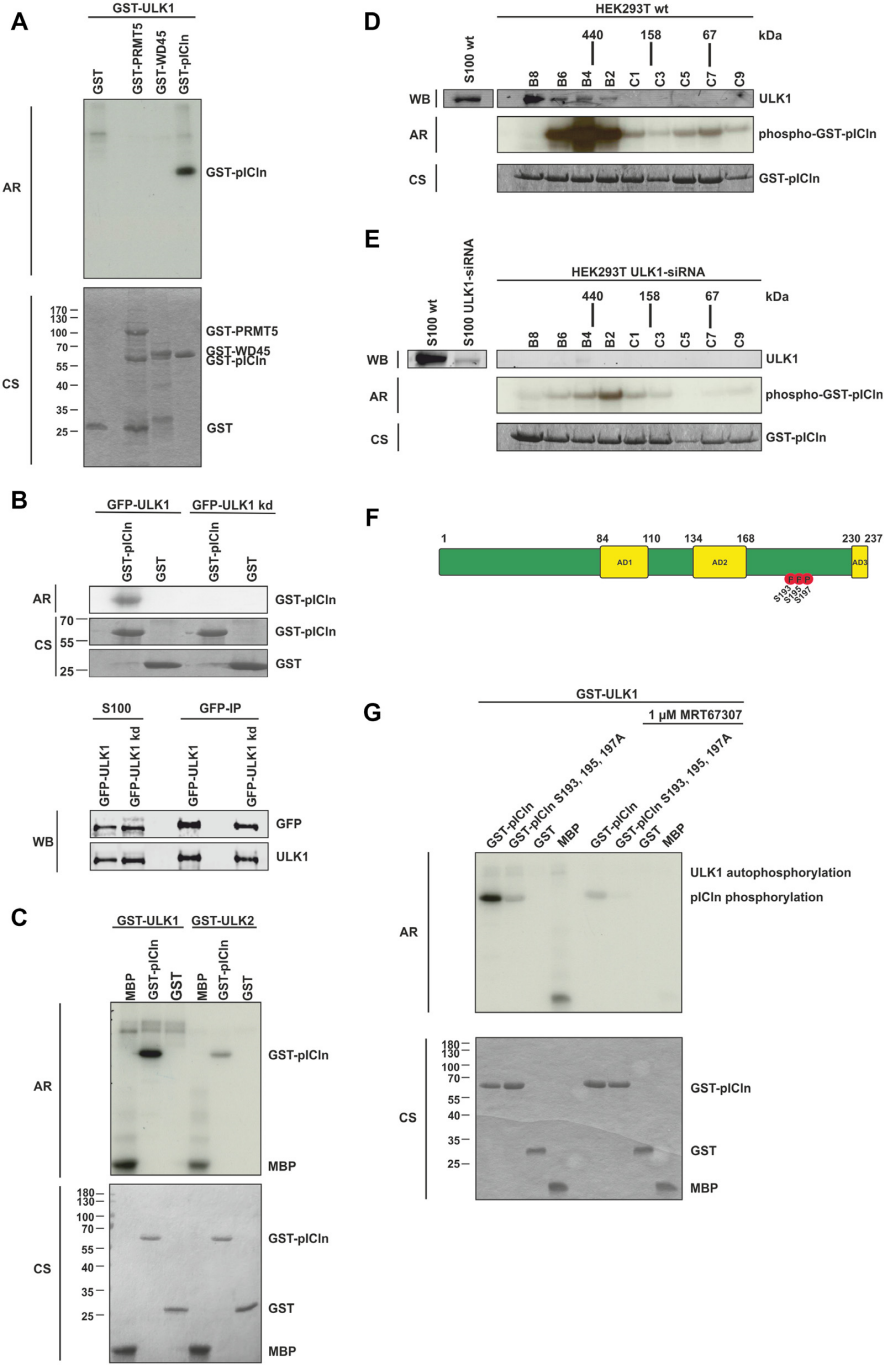
ULK1 phosphorylates pICln in the C-terminal region on residues S193, S195 and S197. (**A**) *In vitro* kinase assay using recombinant active GST-ULK1 expressed in Sf9 insect cells and GST-PRMT5, -WD45, and -pICln purified from *E. coli* as substrate proteins were incubated with 10 μCi [32P]-ATP for 45 min. at 30°C. Samples were separated by Tris/Glycine-SDS-PAGE and analyzed by autoradiography. (**B**) *In vitro* kinase assay with purified GFP-ULK1 or GFP-ULK1 kinase-dead mutant (GFP-ULK1kd) overexpressed in Flp-In T-REx 293 cells and GST-pICln was executed as described in (A). Amounts of the GFP precipitation from GFP-ULK1 and GFP-ULK1 kinase-dead were directly compared by Tris/Glycine-SDS-PAGE and Western-Blot analysis using antibodies against ULK1 and GFP. (**C**) *In vitro* kinase assay using recombinant active GST-ULK1 or -ULK2 expressed in Sf9 cells and GST-pICln was executed as described in (A). (**D**, **E**) Gel filtration was performed with HEK293T wild type (D) and HEK293T ULK1-siRNA knockdown (E) S100 extracts fractionated by a Superdex 200 column and evaluated by western blotting. Appropriate fractions were used for *in vitro* kinase assay using exogenous GST-pICln substrate protein and 10 μCi [32P]-ATP for 45 min. at 30°C and analyzed by autoradiography (Exposure time of 30 minutes for (D) and (E)). (**F**) Schematic view of pICln protein with its three acidic domains (AD1-3) and the ULK1-dependent phosphorylation sites. *In vitro* kinase assay was performed with recombinant active GST-ULK1 from Sf9 cells and GST-pICln as described in (A). After Tris/Glycine-SDS-PAGE and coomassie blue staining, the pICln band was excised and phosphorylation status was analyzed by mass spectrometry (LC-MS/MS). Three phosphosites were detected: S193 (*P* = 3.05E-05, 94.5%), S195 (*P* = 2.3E–07, 100%), S197 (*P* = 2.8E–06, 100%). (**G**) Recombinant active GST-ULK1 from Sf9 insect cells was incubated with 1 μM ULK inhibitor MRT67307 for 30 min at 30°C. *In vitro* kinase assay with inhibitor-treated and non-treated GST-ULK1 was performed using substrate proteins GST-pICln wild type and alanine mutant purified from *E. coli* as described in (A). AR: autoradiography, CS: coomassie blue staining, WB: western blotting. See also Supplementary Figure SD2.

To test if the methylosome is a substrate of ULK1, we performed *in vitro* kinase assays using recombinant purified GST fusion proteins of PRMT5, WD45, and pICln as substrates and active GST-ULK1 purified from Sf9 insect cells. Surprisingly, among all used substrates only pICln showed strong ^32^P incorporation upon incubation with active GST-ULK1 (Figure 2A), indicating that pICln represents a new substrate of ULK1. To prove if the observed phosphorylation of pICln is ULK1 specific, we conducted the kinase assay with a kinase-dead mutant of ULK1 (GFP-ULK1kd) (Figure 2B) (19). Consistent with the previous results, only immunopurified active GFP-ULK1 was able to phosphorylate pICln. In contrast, pICln incubated with the kinase-dead mutant GFP-ULK1kd did not exhibit ^32^P incorporation at all, although expressed and purified in the same amount as ULK1wt (Figure 2B).

Since ULK2 is known to compensate for the activity of ULK1 in the regulation of autophagic processes (33), we investigated whether ULK2 can also phosphorylate pICln. In contrast to ULK1, ULK2 was not proficient to phosphorylate pICln with comparable efficiency (Figure 2C). To evaluate whether ULK1 represents the pICln-associated kinase in cellular extracts we incubated the fractions of a size exclusion chromatography experiment of HEK293T cells with recombinant purified GST-pICln and ^32^P γ-ATP. Autoradiographic analysis of these particular fractions showed strongly phosphorylated GST-pICln in fractions B2, B4 and B6 (Figure 2D). Interestingly, predominant phosphorylation of pICln was observed in fraction B4 that corresponds to the entire PRMT5 complex of ∼440 kDa consisting of all components (PRMT5, WD45, pICln, and ULK1) (SD Figure 2A).

To further confirm the specificity of ULK1 dependent phosphorylation of pICln *in vivo*, we performed siRNA knockdown experiments of ULK1 in HEK293T followed by size exclusion chromatography and subsequent assessment of pICln phosphorylation status by kinase assays using recombinant purified GST-pICln as substrate and ^32^P γ-ATP. By these means, we observed an almost complete reduction in phosphorylation of pICln (Figure 2E), whereas the composition of the PRMT5 complex was not affected (SD Figure 2B). Remarkably, the whole PRMT5 complex migrates in a lower size range if ULK1 is absent (SD Figure 2A, B).

To identify the respective ULK1 phosphorylation sites of pICln, we performed *in vitro* kinase assays following SDS-PAGE, in-gel digestion, and NanoLC–MS/MS analysis. Thereby we identified three novel phosphorylation sites in the C-terminal region of pICln on serine residues 193, 195, and 197 (Figure 2F).

The substitution of all three serines by alanine residues prevented the phosphorylation of pICln by active ULK1 (Figure 2G). Also, pharmacological inhibition of active ULK1 with the ULK1/ULK2 specific inhibitor MRT67307 (34) significantly reduces phosphorylation of pICln (Figure 2G). MS analyses of phosphorylation of pICln by ULK1 showed exclusive phosphorylation of the C-terminal domain. In this region, serines 193,195, and 197 appeared well ahead of other putative phosphorylation sites with approximately equal signal strength. Many ULK1 substrates have multiple ULK-specific phosphorylation sites of serines, such as ATG13, FIP200, Beclin1, and AMBRA (35). This prompted us to investigate the ULK-specific phosphorylation of pICln at individual serine residues 193, 195, and 197. However, no preference was detected in *in vitro phosphorylation* experiments in which these residues were replaced individually (SD Figure 2C, D). Random single phosphorylation or patch phosphorylation appears to be the more favorable model in the case of pICln with respect to known ULK1 substrates. Taken together, we demonstrated by amino acid exchange within pICln as well as ULK1 specific inhibition, ULK1 siRNA, and ULK1 kinase-dead mutant that ULK1 binds and phosphorylates pICln on newly identified sites within its C-terminal region.

### ULK1 dependent phosphorylation of pICln regulated binding of pICln towards SmG

**Figure 3.**
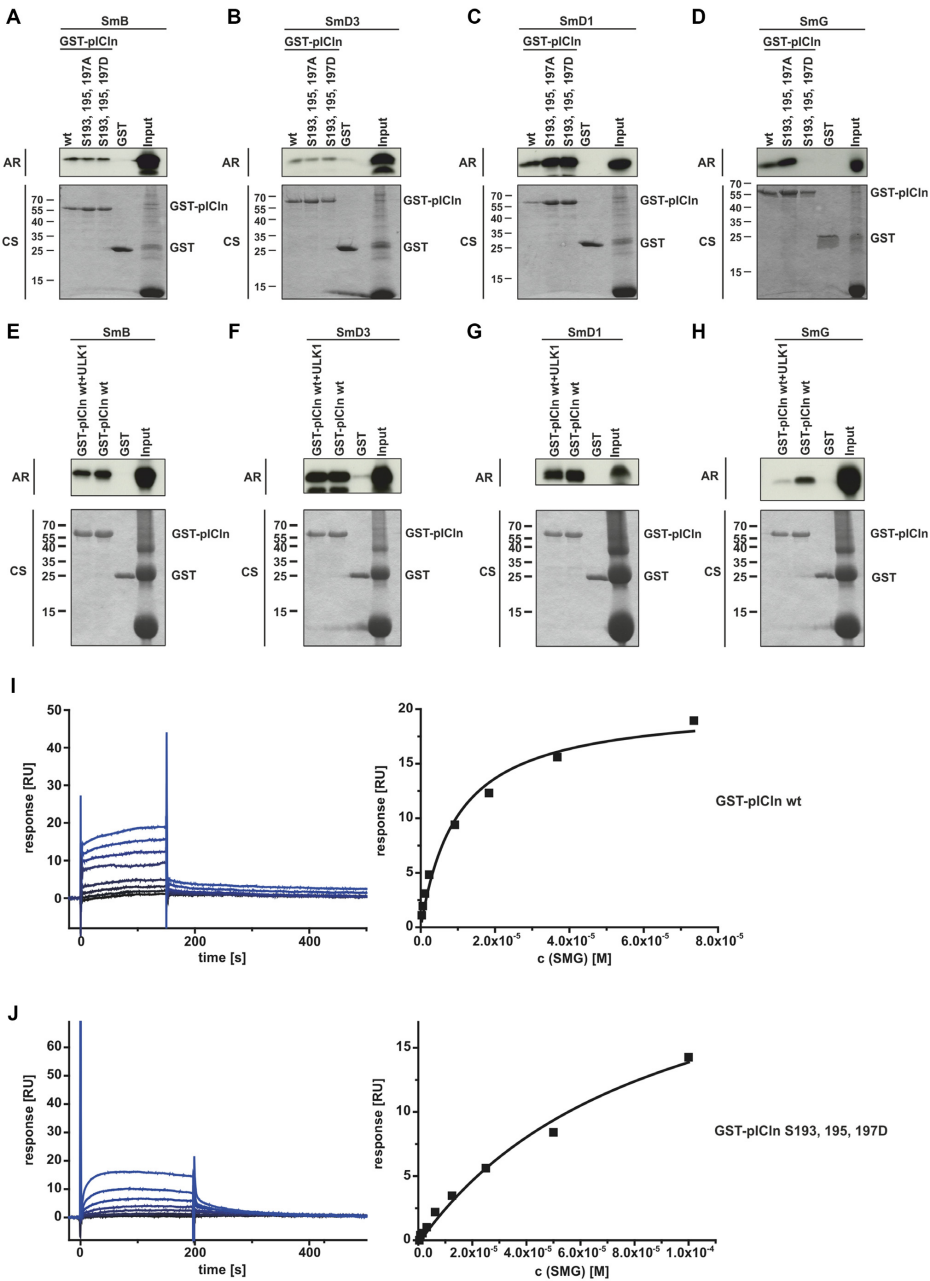
ULK1 dependent phosphorylation of pICln regulated binding of pICln towards SmG. (**A–D**) *In vitro* translated L-[35S]-Methionine labelled Sm Proteins D1, D3, B, and G were applied to an interaction assay with GST-pICln wt and phosphomutants purified from *E. coli*. After incubation for 1.5 h at 4°C and two times washing, purified proteins were separated by Tris/Glycine-SDS-PAGE and analyzed by autoradiography. (**E-H**) *In vitro* translated L-[35S]-Methionine labelled Sm Proteins D1, D3, B, and G were applied to an interaction assay with GST-pICln wt and GST-pICln pre-phosphorylated by ULK1. Pre-phosphorylation of pICln was performed for 1.5 h with 100 ng of active ULK1. After incubation for 1.5 h at 4°C with translated Sm Proteins and two times washing, purified proteins were separated by Tris/Glycine-SDS-PAGE and analyzed by autoradiography. (**I**, **J**) SmG (analyte), and GST-pICln (ligand) wild type, and aspartate mutant were purified from *E. coli*. Affinity dissociation constants were analyzed by surface plasmon resonance by steady-state analysis (SmG – GST-pICln wt, *K*_d_ 11.5 ± 1.5 μM; SmG – GST-pICln S193,195,197D, *K*_d_ 84.7 ± 14.7 μM; *n* = 3). AR: autoradiography, CS: coomassie blue staining.

pICln is part of the methylosome complex (5,7). For this, we investigate to what extent phosphorylation of pICln does affect PRMT5-mediated methylation of Sm proteins. Hence, we performed *in vitro* methylation assays using the substrate proteins SmB, D1, and D3 with or without the presence of pICln wildtype and phosphomutants thereof. For the methylation of SmB and D3, the presence of pICln does not seem to be necessary, confirming so far findings by Neuenkirchen and colleagues (36). This is in clear contrast to SmD1, as SmD1 is only methylated in the presence of pICln. However, comparing the methylation efficiency of Sm proteins, we see no difference using pICln wildtype or the phosphomutants thereof (SD Figure 3A and B). To this end methylation of SmD1 is pICln-dependent but independent of the phosphostatus of pICln (SD Figure 3B).

Extensive and conclusive analysis by A. Chari and coworkers revealed the function of pICln as an assembly chaperone in the biogenesis of UsnRNPs (13). In this function, pICln forms two subcomplexes: One consisting of pICln, the Sm proteins B and D3 (SmB/D3) and the other subcomplex consists of pICln and the Sm proteins D1, D2, E, F and G (SmD1/D2/E/F/G), see also Figure 8). In the latter case pICln directly binds to SmD1 and G and builds a highly stable hexameric ring structure, also known as the 6S complex (5,13,37). Consequently, we investigated to which extend the phosphorylation status of pICln affects its binding to the Sm proteins B, D3, D1, and G. To this end, we used GST-tagged recombinant pICln and phosphomutants thereof to analyze the interaction with *in vitro* translated ^35^S-labelled SmB, SmD3, SmD1 and SmG (Figure 3A–D). Neither the phospho-inactivating serine-to-alanine nor the phospho-mimicking serine-to-aspartate substitutions at positions 193, 195, and 197 affected the binding capacity of pICln to the Sm proteins B, D3 and D1 (Figure 3A–C). In striking contrast, the phospho-mimicking aspartate mutations of pICln displayed no interaction with SmG, whereas the phospho-inactivating alanine mutations of pICln exhibited increased binding to SmG (Figure 3D).

In addition to these results binding of GST-pICln wildtype, phosphorylated by ULK1 does not affect association to the Sm proteins B, D3, and D1 (Figure 3E–G). In contrast, pICln, phosphorylated by ULK1 shows dramatically reduced interaction with SmG compared to unphosphorylated pICln (Figure 3H).

These results were further corroborated by surface plasmon resonance measurement. The affinity of wild-type pICln for SmG yielded a dissociation constant (*K*_d_) of 11.5 ± 1.5 μM (Figure 3I). The introduction of phospho-mimicking aspartate mutations (S193, 195, 197D) within pICln reduced the affinity for SmG by almost one order of magnitude to a *K*_d_ of 84.7 ± 14.7 μM (Figure 3J).

Summarizing results so far, we see that phosphorylation of pICln in its C-terminal part by ULK1 does not alter binding properties to SmD1 but does block its binding towards SmG.

### Phosphorylation of pICln by ULK1 alters the structure of the 6S complex

**Figure 4.**
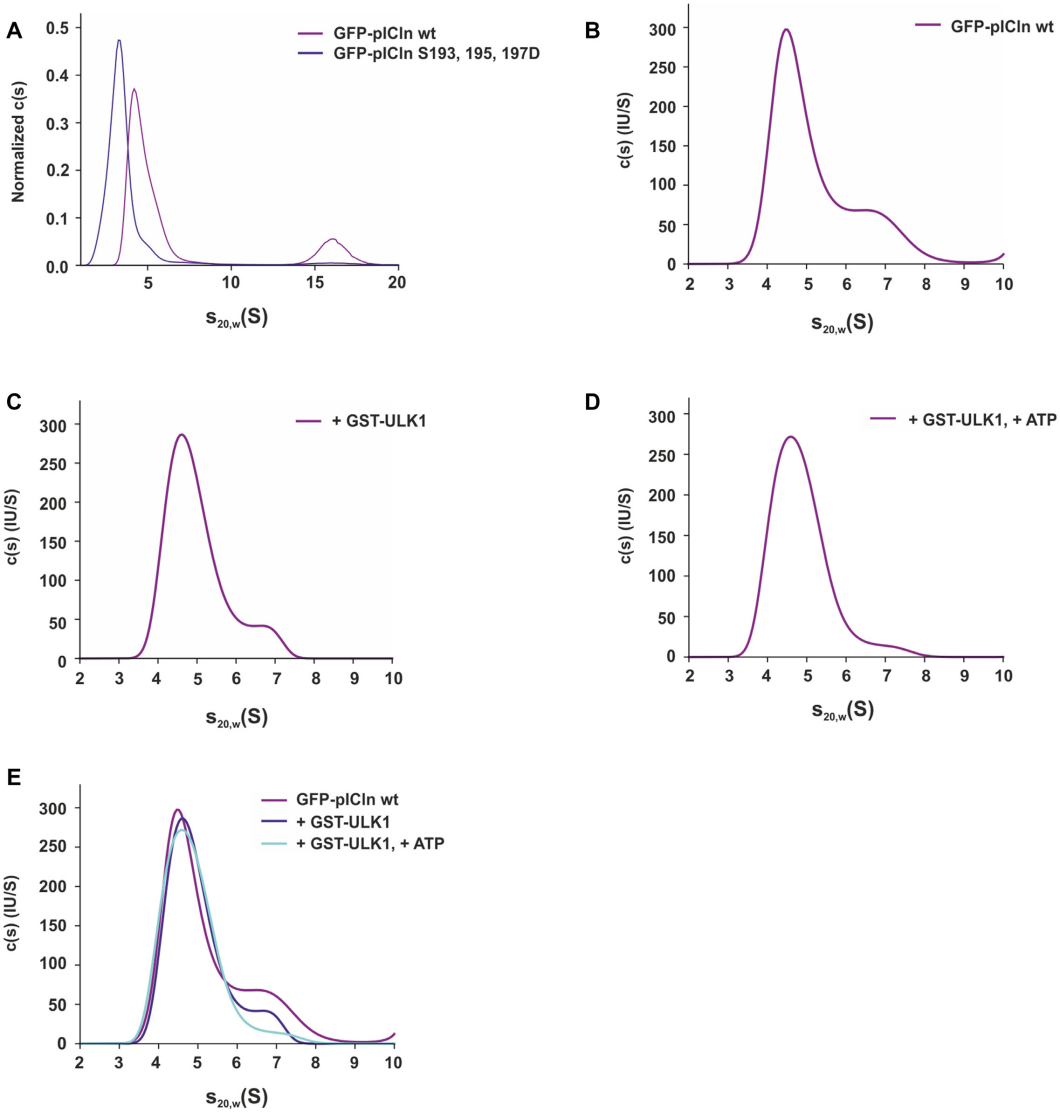
Phosphorylation of pICln by ULK1 alters the structure of the 6S complex. (**A**) Sedimentation velocity (SV) analysis of GFP-pICln wild type and aspartate mutant in S100 extracts. The c(s) distributions for wild type pICln complex (magenta curve) and pICln complex with S193, S195, S197D mutations (blue curve) obtained from SV analysis at 40 000 rpm at 20°C for 5 h are shown. For better comparability normalization according to the area under the curve was done. (B–E), Size exclusion chromatography of Flp-In T-REx 293-GFP-pICln S100 extract using a Superdex 200 increase column was performed following a sedimentation velocity analysis (**B**) for GFP-pICln wild type of the 6S fractions B13 and B12. (**C**) Sedimentation coefficient distribution for wild-type pICln complex incubated with recombinant active GST-ULK1. (**D**) Sedimentation coefficient distribution for wild-type pICln complex incubated with recombinant active GST-ULK1 and ATP. (**E**) Overlay of *c*(*s*) distributions for all three samples acquired under the same conditions. All measurements were performed at 40 000 rpm at 20°C for 5 h. See also Supplementary Figure SD3.

These findings are in line with Grimm and colleagues, who proposed the pICln-SmG contact surface as a ‘mobility hotspot’ of the 6S structure by *in silico* prediction (38). From their comprehensive work, the authors concluded that the 6S ring structure has to be transiently opened on the pICln-SmG interface to be able to load Sm proteins onto the SMN complex. To evaluate if phosphorylation of pICln within the 6S complex also alters its composition or structure *in vivo* we used analytical ultracentrifugation (AUC). AUC analysis is an absolute method to determine the size and shape of macromolecules in solution. The sedimentation of macromolecules in a centrifugal force field depends on molecular mass, as well as shape according to the Svedberg equation (SV) (39). SV analysis was applied to detect potential differences in the conformations of wild-type pICln complex and pICln complex with S193, S195, S197D mutations. The used GFP tagged phosphomutants of pICln can interact with its well-known binding partners PRMT5, WD45 and SMN to the same extent as wildtype pICln does (SD Figure 3A). Also, no differences in interaction with Sm proteins or methylation efficiency could be detected (SD Figure 3A and B), ensuring the functionality of used mutated constructs. A closed ring structure is expected to sediment faster than an elongated open ring structure of the same mass because of less friction. For this experiment, a GFP variant of pICln was used. This specific labelling allowed tracing of pICln in its diverse states during ultracentrifugation, using SV-AUC equipped with a fluorescence detection system, in the presence of cytoplasm extract. Other non-fluorescing proteins or macromolecules also present in the S100 extract are invisible. As shown in Figure 4A, the SV analysis of the wild-type complex revealed a major species with a weight averaged *s*_app_-value of 4.7 S and a minor species at 16.1 S. For the mutated complex, however, only one species with a weight averaged *s*_app_-value of 3.5 S was detected. Concomitantly, the weight average frictional ratio (*f/f*_0_), which is informative on the hydrodynamic shape of a molecule in solution, was higher in the c(*s*) analysis for the serine-to-aspartate mutant pICln complex. Assuming that the molar mass of the complex stays constant during centrifugation, the SV analysis suggests that the mutant form of the complex has a different, more elongated conformation than the wild-type complex. Since the density and viscosity of the cytoplasm extract were unknown only the relative differences between wild type and mutant complex can be reported as apparent sedimentation coefficients *s*_app_. These results are in agreement with our model of a closed ring conformation for the wild type and an open ring structure for the serine-to-aspartate mutant pICln-complex (for the model also see Figure 8).

To examine whether the phosphorylation of pICln leads to a conformational change of the 6S complex and thus to a reduced sedimentation velocity, we purified the 6S complex containing GFP-pICln by size exclusion chromatography (pooled fractions B12 and B13, SD Figure 3C) and incubated it with active GST-ULK1 and ATP. As a control, we included the active kinase ULK1 but no additional ATP to demonstrate that the observed effects are not due to the presence of the kinase only but require phosphorylation of pICln. As can be seen from Figure 4B the wild-type pICln complex displayed a heterogeneous distribution pattern in our experiment, with a major peak at 4.5 S and a shoulder peak at about 6.3 S. A very similar distribution was observed in the pICln complex incubated with ULK1 (Figure 4C). However, the addition of ATP to pICln complex with ULK1 significantly reduced the fraction of the species at ∼6.3 S, as depicted in Figure 4D, suggesting that the closed-ring structures have been converted to open-ring structures in the presence of ULK1 and ATP.

The measured *s*-value of the complex in the present study is in agreement with the literature for the 6S ring-shaped complexes (38). The SV analysis demonstrated that the phospho-mimicking pICln complex is indeed more elongated than the wild-type complex (Figure 4A). Additionally, we could show that phosphorylation of pICln within the wild type 6S complex by ULK1 favors the formation of open ring structures (Figure 4B-E).

### Phosphorylated pICln is not able to build the 6S complex and to promote the subsequent Sm protein transfer onto the SMN complex

**Figure 5.**
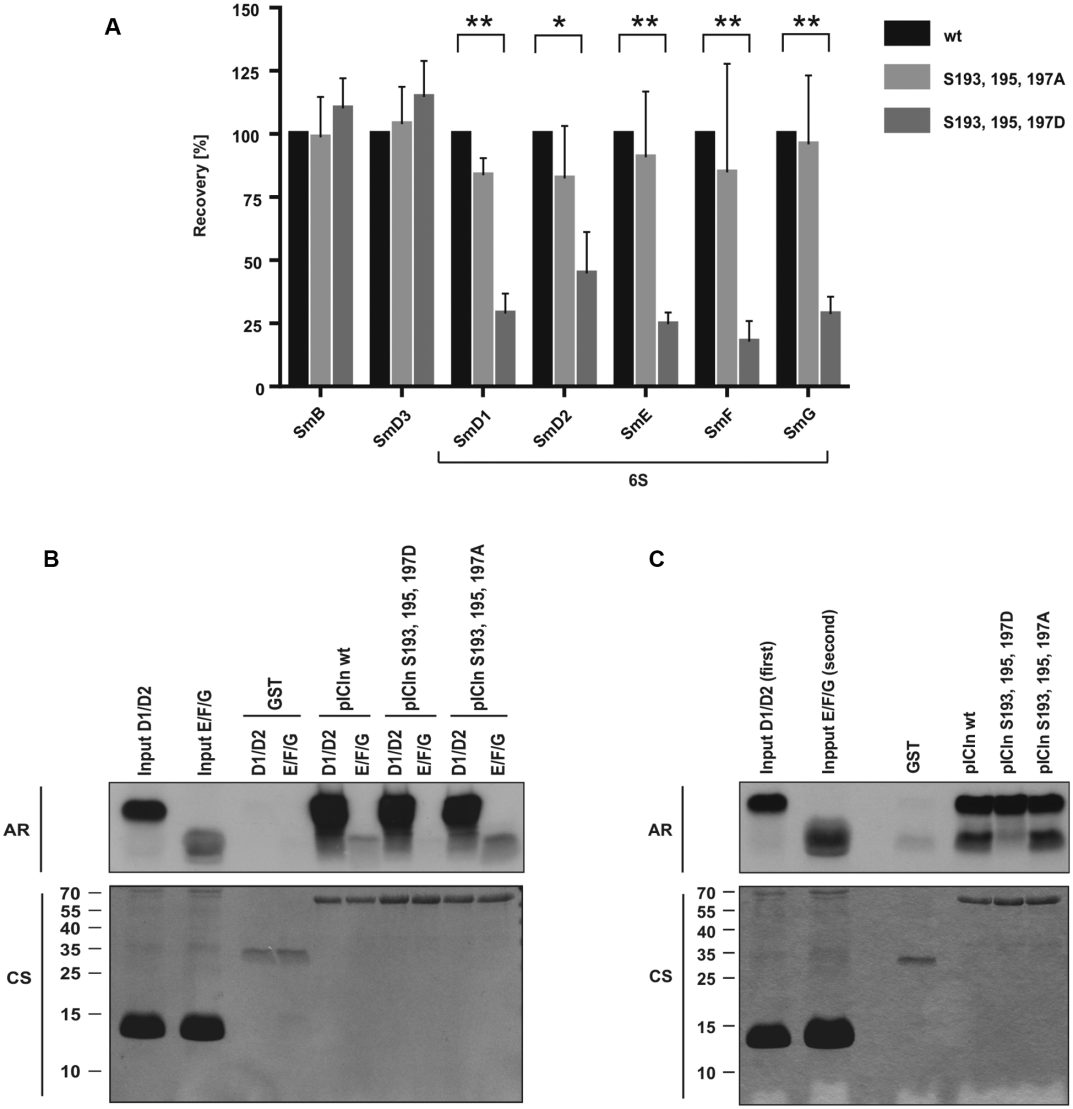
Phosphorylated pICln is not able to build the 6S complex and to promote the subsequent Sm protein transfer onto the SMN complex. (**A**) Pulldown experiments using GST-pICln wild type and phosphomutants were executed in HEK293T S100 extract overnight at 4°C and co-purified Sm proteins were quantified by mass spectrometry and normalized to pICln wild type (LC-MS/MS; *P < 0.05; **P < 0.01). (**B**) Sm proteins D1, D2, E, F and G were *in vitro* translated and labelled with l-[35S]-Methionine. D1/D2 and E/F/G were pre-incubated for 1 h at 30°C and applied to an interaction assay with GST-pICln wt and phosphomutants purified from *E. coli*. Following incubation for 1.5 h at 4°C and three times washing purified proteins were separated by Tris/Glycine-SDS-PAGE and analyzed by autoradiography. (**C**) Sm proteins D1, D2, E, F and G were *in vitro* translated and labelled with L-[35S]-Methionine. To form the 6S structure D1/D2 and E/F/G were pre-incubated for 1 h at 30°C. To assess the influence of pICln as an ‘assembly chaperone’ Sm protein complex D1/D2 was first incubated (1 h at 4°C) using GST-pICln wt or phosphomutants purified from *E. coli*. After three times washing of the resulting pICln–SmD1/D2 complex, SmE/F/G complex was added to the mixture for a further 1 h at 4°C. After three times washing purified proteins were separated by Tris/Glycine-SDS-PAGE and analyzed by autoradiography. AR: autoradiography, CS: coomassie blue staining.

To prove whether the phospho-dependent interaction between pICln and SmG is crucial for the biogenesis of the 6S complex *in vivo*, we performed pulldown assays with recombinant GST-tagged wild type, phospho-mimicking (S193, 195, 197D) and -inactivating (S193, 195, 197A) pICln proteins in HEK293T derived cytosolic extracts. Pulldowns were followed by protein identification and relative protein quantification by mass spectrometry analysis (for details see materials and methods section). Thus both, the phospho-mimicking and -inactivating pICln mutants pulled down the same amount of the SmB/D3 subcomplex normalized to pICln wild type (Figure 5A). In contrast, all components of the 6S complex (SmD1/D2/E/F/G) displayed a substantial reduction in binding to phospho-mimicking pICln mutant whereas the binding capacity of the phospho-inactivating pICln mutant was only slightly affected (Figure 5A). These data underscore that the phosphorylation status of pICln represents a crucial event in the assembly of the 6S complex: phosphorylated pICln is not able to bind SmD1/D2/E/F/G to the same extent as non-phosphorylated pICln.

Neuenkirchen *et al.* postulate a model in which pICln binds first to SmD1 and D2 and recruits it for methylation by PRMT5/WD45. After methylation of SmD1 pICln further recruits the subcomplex of SmE/F/G to assemble the final 6S complex that stays associated with the PRMT5 complex (36). Beyond that, we could now show that ULK1 interacts with the PRMT5/6S complex and phosphorylates the C-terminus of pICln. To test the influence of ULK1 mediated pICln phosphorylation towards the biogenesis of the 6S complex, we executed direct binding studies. For this, we used GST-tagged recombinant pICln and phosphomutants thereof to investigate the interaction with *in vitro* translated ^35^S-labelled preformed Sm protein subcomplexes D1/D2 and E/F/G (Figure 5B and C). Neither the serine-to-alanine nor the phospho-mimicking serine-to-aspartate substitutions at positions 193, 195 and 197 affected the binding capacity of pICln to the SmD1/D2 subcore (Figure 5B). In contrast, the phospho-mimicking aspartate mutant of pICln did not interact with the SmE/F/G subcore in comparison to the serine-to-alanine mutant and the wild-type protein (Figure 5B). Next, we reconstituted the entire 6S complex in a stepwise process, using *in vitro* translated ^35^S-labelled Sm proteins. In a first step, we incubated GST-tagged recombinant pICln or phosphomutants with *in vitro* translated ^35^S-labeled subcomplex SmD1/D2. In a subsequent step and after intensive washing, we added *in vitro* translated ^35^S-labeled subcomplex SmE/F/G. Consistent with our previous data Sm proteins E/F/G only bind to the pICln-SmD1/D2 subcomplex with the wild-type protein and the serine-to-alanine mutant of pICln, but not to the pICln-SmD1/D2 subcomplex with the serine-to-aspartate mutant (Figure 5C). We also observed that the binding of the SmE/F/G subcomplex to pICln wt and alanine mutant was increased after incubation with the SmD1/D2 subcomplex (Figure 5B and C), supporting the model of a sequential binding of Sm protein subcomplexes during 6S assembly.

Our results demonstrate that binding of single Sm proteins, Sm-subcomplexes as well as the formation of a reconstituted entire 6S complex depend on the phosphorylation status of the C-terminus of pICln and its interaction with SmG.

### Inhibition of ULK1 results in a decreased number of Cajal bodies

**Figure 6.**
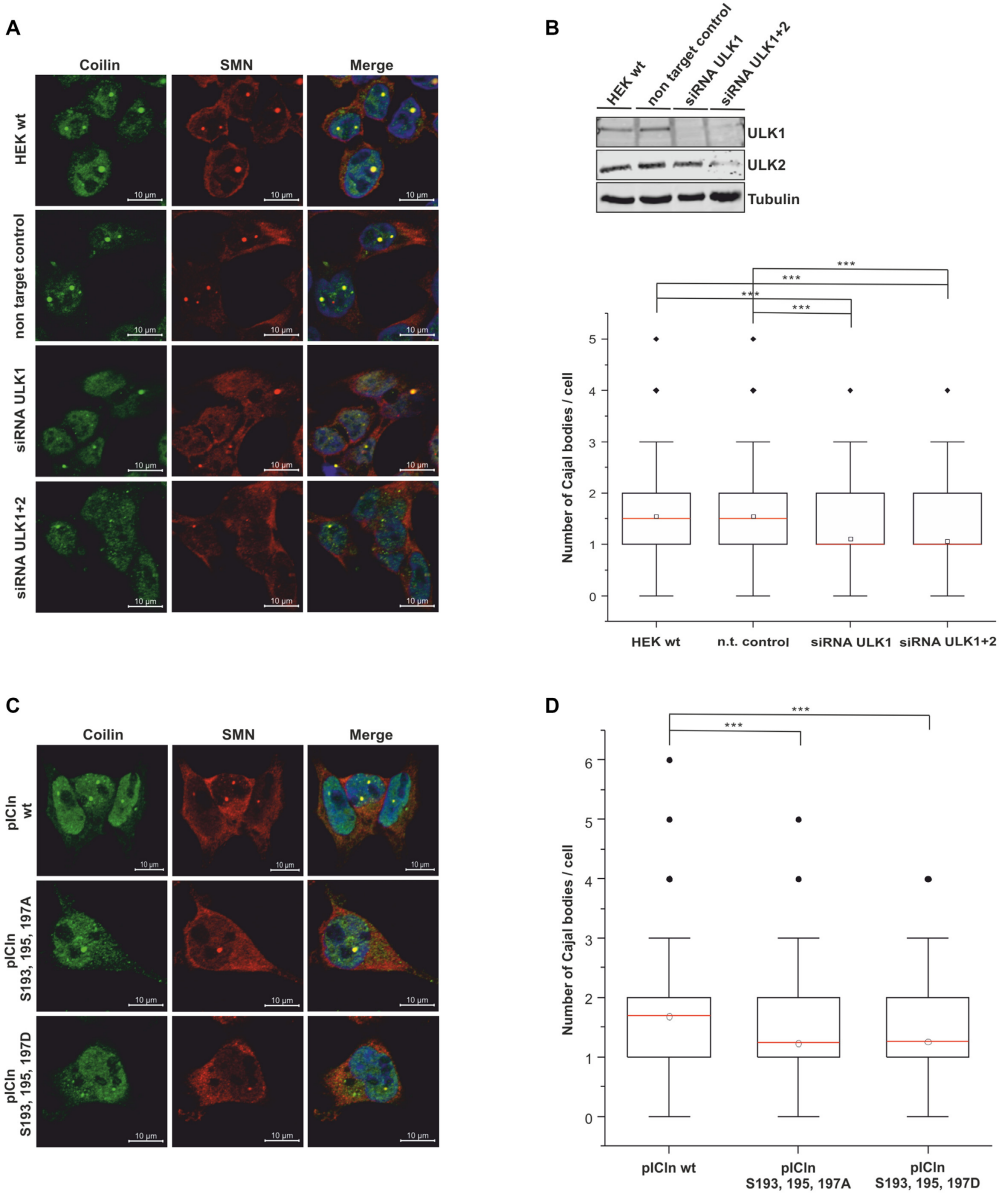
Decrease of endogenous ULK1 results in a decreased number of Cajal bodies. (**A**, **B**) HEK293T cells were treated with 50 nM ULK1,2 siRNA or non-targeting control for 48 h. (**A**, **C**) The cells were fixed and Cajal bodies were visualized with antibody staining against Coilin (green) and SMN (red). The DNA was stained with DAPI (blue). (**B**) Cell lysates of siRNA-treated cells were analyzed by Tris/Glycine-SDS-PAGE using antibodies against ULK1, ULK2, and Tubulin. The downregulation of ULK1 causes a reduction in the snRNP storage pool. In the boxplot diagram, the ‘box’ represents 25–75% of all values and the mean (red), standard deviation, and out layers are visualized. HEK293T cells show an average of 1.54 (n = 500) Cajal bodies. Treatment of cells with siRNA caused a 1. significant decrease in the number of Cajal bodies. (**C**, **D**) The phosphorylation status of pICln influences the number of Cajal bodies per cell. (**D**) Overexpression of pICln in Flp-In T-Rex cells causes an increase in the number of Cajal bodies, mean 1.69 (n = 502). Phosphomutants of pICln (S193, 195, 197A, or D), cause a decrease in the number of Cajal bodies per cell. The *P*-value was calculated with Origin using the Mann–Whitney *U* test. ***P < 0.005; scale bars: 10 μm (A and C).

Since Cajal (CBs) bodies dynamically form as a self-organized system, i.e. whenever the concentration of important components or macro-complexes reaches a concentration threshold (40), inhibition of snRNP biosynthesis should lead to a reduction of CBs. Therefore we treated HEK293T cells with ULK1 and ULK2 siRNA followed by immunofluorescence using SMN and Coilin specific antibodies to visualize the quantity of Cajal bodies as a marker of snRNP assembly capability (40–42) (Figure 6A, B). The number of CBs per nucleus (mean) was significantly decreased in ULK1 knockdown HEK cells (1.10) compared to HEK control cells (1.54) without cumulative effect by concurrent knockdown for ULK2 (1.10 compared to 1.06) (Figure 6A, B). These results were confirmed by treatment of cells with the ULK1 inhibitor MRT67307 (SD Figure 4A). The number of CBs per nucleus (mean) was significantly decreased in HEK293T cells after 3 h (0.78) and 5 h (0.6) upon inhibitor treatment compared to HEK cells without treatment (1.49) (SD Figure 4B).

Comparable results were obtained when we investigated the number of CBs in HEK cells overexpressing phospho-mimicking (S193, 195, 197D) and -inactivating (S193, 195, 197A) pICln proteins (Figure 6C, D). The mean of CBs in HEK Flp-In T-Rex cells overexpressing the pICln phosphomutants was decreased (1.22 for the alanine mutant and 1.26 for the aspartate mutant) compared to 1.64 in HEK Flp-In T-Rex cells overexpressing the wildtype protein (Figure 6C, D). In summary, the immunostaining results clearly show an altered content of UsnRNPs upon ULK1-dependent phosphorylation of pICln in the cell.

### ULK1 regulates UsnRNP biogenesis

**Figure 7.**
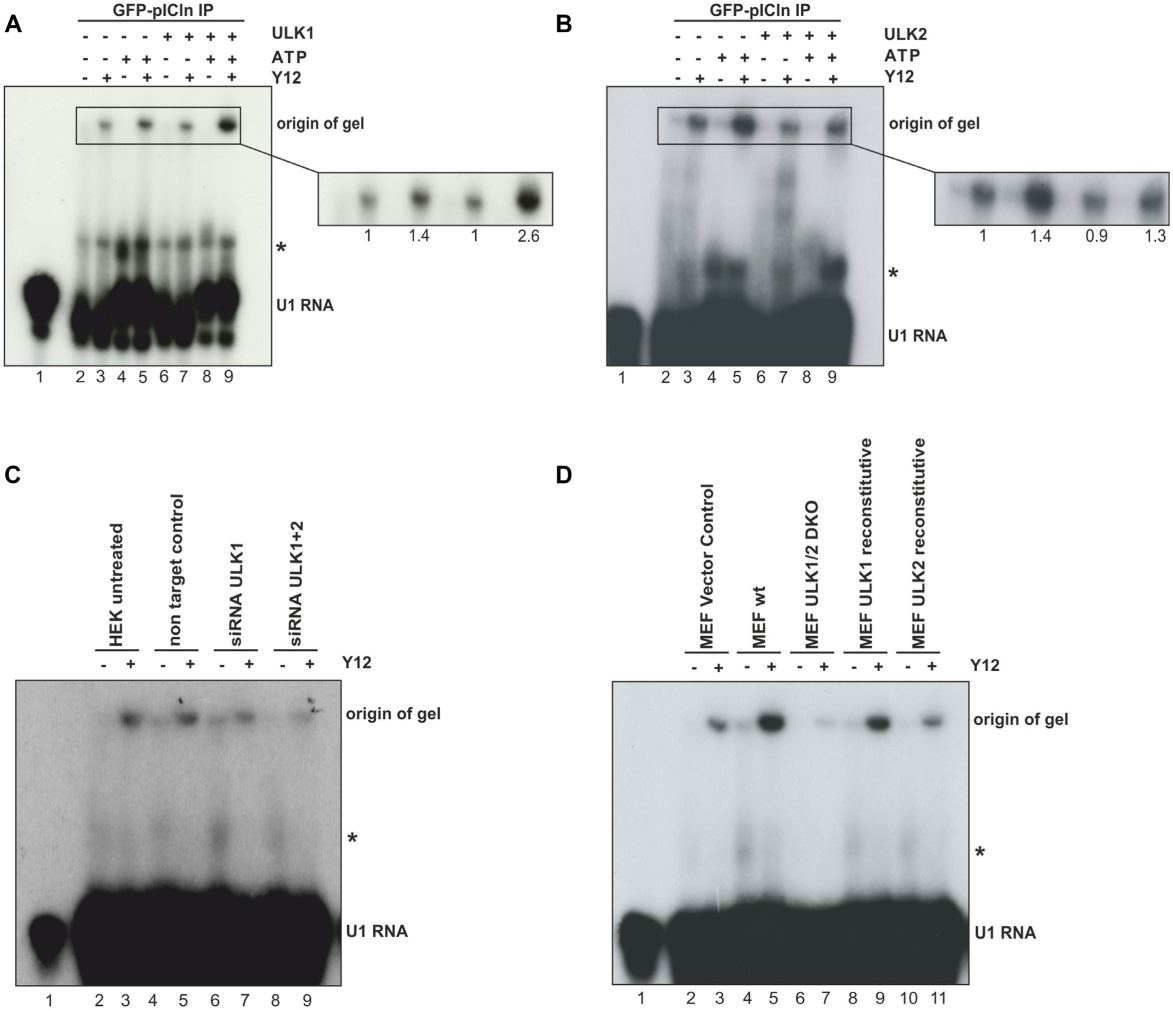
ULK1 phosphorylation of pICln regulates UsnRNP biogenesis. (**A**, **B**) GFP-pICln IP contains all proteins necessary for U1 snRNP core assembly. *In vitro* transcribed U1 snRNA labeled with 10 μCi [32P]-UTP was incubated with GFP-pICln IP. After incubation samples were directly analyzed by native gel electrophoresis (2, 4, 6, 8), or the same samples were subjected to supershift analysis with the Y12 antibody to show the specific formation of snRNPs (3, 5, 7, 9). The formation of snRNPs was quantified with Image Studio to compare the efficiency of the snRNP biogenesis. Adding ATP to the GFP-pICln IP increases efficiency (1.4) while adding ATP and ULK1 (2.6) leads to the highest efficiency in snRNP formation compared to the GFP-pICln IP alone. Adding ULK2 and ATP to the GFP-pICln IP caused no effect at all (1.3). (**C**) *In vitro* transcribed U1 snRNA labelled with 10 μCi [32P]-UTP was incubated with S100 extract from HEK293T cells treated with siRNA against ULK1, ULK 1+2, or a non-targeting control. After a native gel electrophoresis supershift analysis was performed using the Y12 antibody. (**D**) S100 extract from Mouse Embryonic Fibroblasts (MEFs) lacking ULK1/2, or reconstituted with vector control, ULK1 or ULK2 were incubated with [32P] labelled U1 snRNA, after a native gel electrophoresis supershift analysis occurred.

As the phosphorylation status of pICln influences the nuclear Cajal bodies, we asked whether ULK1 directly regulates the UsnRNP assembly activity. By using native polyacrylamide gel electrophoresis, we demonstrate that immobilized GFP-pICln derived from HEK Flp-In T-Rex cells can generate U1snRNP cores super-shifted by the anti-Sm antibody Y12 upon incubation with a human *in vitro* transcribed ^32^P-labelled U1snRNA (Figure 7A, compare lanes 2 and 3). This assembly reaction is remarkably increasable by adding ATP (Figure 7A compare lanes 3 and 5) confirming the ATP dependency of the UsnRNP biogenesis by previous studies (8,14,18). It also implies that the corresponding kinase is associated with the immunoprecipitated complex, capable of UsnRNP assembly. The addition of purified ULK1 to the immobilized GFP-pICln/U1 snRNA mixture without extra ATP does not affect the assembly efficiency (Figure 7A, compare lanes 3 and 7). In striking contrast, the simultaneous addition of ULK1 and ATP strongly increases the ability of U1 snRNP core biogenesis (Figure 7A, lane 9). UsnRNP assembly efficiency increases up to 2.6-fold upon the addition of ATP and active ULK1 (see enlarged part of Figure 7A). On the contrary, the addition of active ULK2 has no stimulatory effect on the UsnRNP assembly efficiency (Figure 7B).

**Figure 8.**
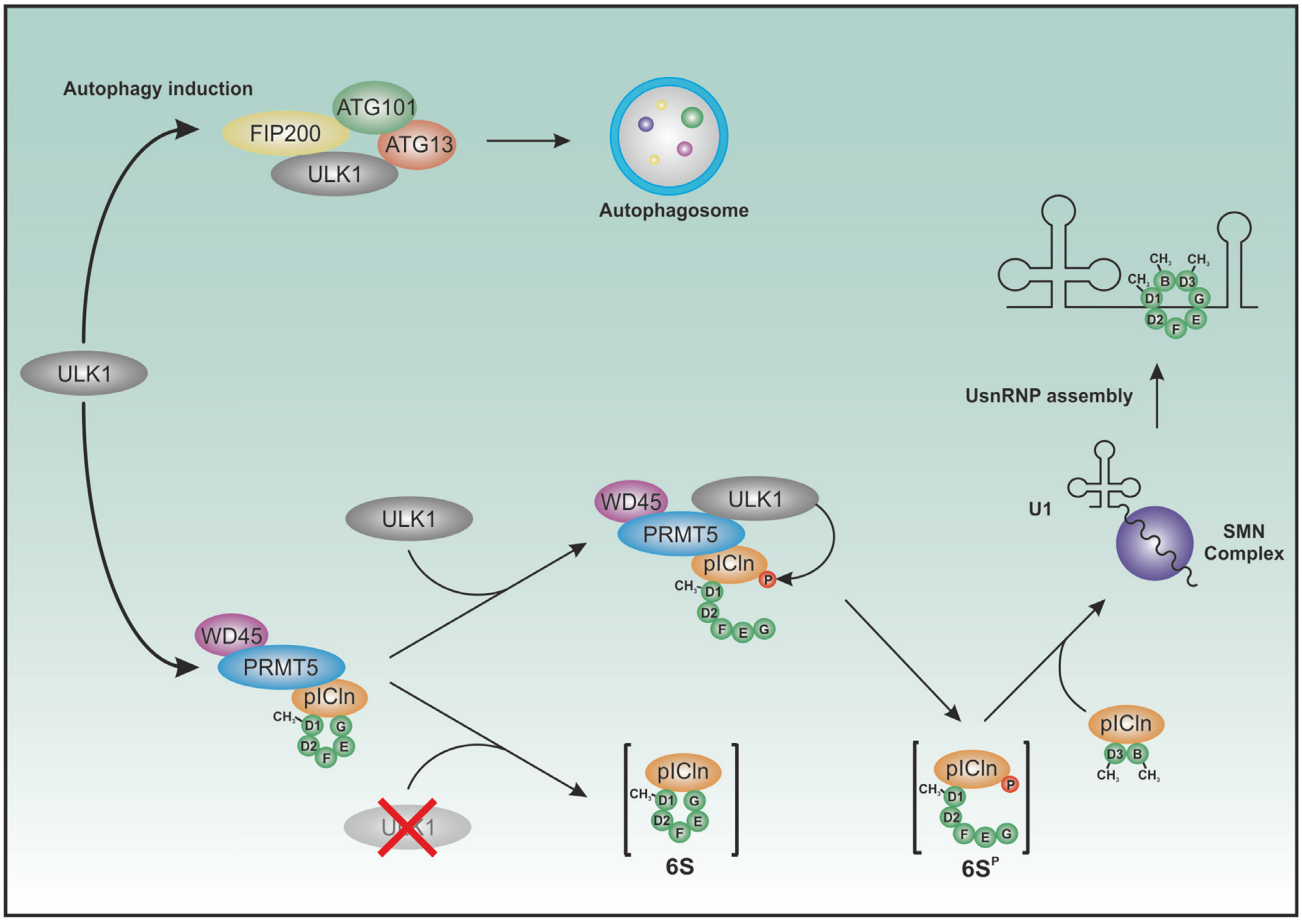
Schematic illustration summarizing the new role of ULK1 in the UsnRNP assembly as well as its well-known function in autophagy induction.

Complementary to these results HEK cells treated with siRNA against ULK1 and ULK1/ULK2 shown reduced assembly UsnRNP assembly activity (Figure 7C). A comparable reduction was observed in constitutive ULK1/ULK2-double knockout MEF cells (Figure 7D). Only double-knockout cells reconstituted with ULK1 but not ULK2 alone can assemble snRNPs with comparable efficiency as MEF wild-type cells do (Figure 7D).

This data demonstrates that an isolated, purified assembly complex directly is capable to increase UsnRNP biogenesis upon the addition of ATP. It further clearly shows that only ULK1 stimulates UsnRNP biogenesis by direct phosphorylation of pICln. To test whether the observed snRNP biogenesis effects are ULK1-dependent and not due to an overall autophagy-dependent effect we used ATG3 knockouts MEF cells (SD Figure 5A). Autophagy is completely blocked in this cell line (43) (SD Figure 5A). However, we could detect any difference concerning snRNP assembly activity between normal or ATG3 knockout cells (SD Figure 5B).

## DISCUSSION

Although the ATP dependency of the Sm core assembly and phosphorylation of some key components within the UsnRNP pathway has been known for many years (8,15,16,44), neither the responsible kinases nor the structural or mechanistic consequences of the modified residues were known so far. In our study, we identified the Ser/Thr kinase ULK1 as a functional component of the PRMT5 complex and as an essential key regulator of UsnRNP biogenesis by specific phosphorylation of pICln.

The identified phospho-serines 193, 195 and 197 are located in a region of pICln that, based on crystal structures, is not known for Sm protein binding so far, but match the previously reported consensus sequence for ULK1 phosphorylation (35). Certainly, the structure determination of pICln has only been executed with pICln of *D. melanogaster*, C-terminal deletions of pICln of *D. melanogaster* (13,38,45), or with the isolated N-terminus of canine pICln (46,47). Recent studies also have determined the C-terminus of canine pICln by NMR (48). The authors demonstrated that the C-terminus of pICln is highly conserved in vertebrates and natively unstructured with secondary structure elements. These unstructured regions often function as flexible linkers in the assembly of macromolecular systems regulated by post-translational modifications (49). The lack of essential elements of the intrinsic disorder region in initial experiments based on *D. melanogaster* pICln may explain why this region was not addressed for Sm protein binding in the human system so far (SD Figure 6) (50). It also may explain the difficulties to solve the crystal structure of the human 6S intermediate complex, containing flexible unstructured regions (45). In the capacity of an assembly chaperone of Sm proteins (13), the conformational flexibility of pICln generated by the disordered C-terminus is an important property in the consecutive transfer of the Sm proteins onto the SMN complex. The work presented here specifically does focus on the exclusive use of human proteins and human snRNA to assess the status and mechanism of human Sm core assembly and UsnRNP biogenesis.

The newly identified ULK1-dependent phosphorylation sites within the C-terminus of pICln regulate the contact surface of pICln and SmG (Figures 2F and 3D, H-J); consequently, they also influence binding properties towards the SmE/F/G subcomplex (Figure 5B, C). Consistent with these are the results from the analytical ultracentrifugation analysis, demonstrating that the phosphorylation of purified cytoplasmic 6S complex by ULK1 favors the formation of an open ring structure (Figure 4E). As the formation of 6S, along with UsnRNP assembly is a sophisticated process also involving the interaction of 6S with the PRMT5- and subsequently, also the interaction with the SMN complex, re-arrangements on PRMT5-6S-SMN cannot be excluded completely. Therefore, it would also be conceivable that the phosphorylation state of pICln impairs the transfer of Sm proteins to the SMN or from the PRMT5 complex. Direct structural data by Cyro-EM or NMR upon phosphorylation of pICln finally will cover the dynamics of this process on a molecular level in the future.

These results prove the pICln-SmG contact surface as a mobility hotspot of the 6S complex (38). Our data also provide information on the composition and conformation of the native human 6S complex and its functional regulation by phosphorylation of pICln.

We further show that ULK1 dependent phosphorylation sites within the C-terminus of pICln affect the interaction between pICln and SmG. Recent studies convincingly demonstrated that the SMN subcomplex consisting of SMN and GEMIN2 can directly bind the Sm pentamer via GEMIN2 (9). The authors pointed out that there probably exist at least two sequential occurring mechanisms of Sm pentamer binding: A first step by binding to pICln, as a second sequential one by binding to GEMIN2. Future studies of pICln/phospho-pICln and the SMN/SMN subcomplexes are necessary to address the detailed mechanism of how phosphorylated pICln contributes to this step of UsnRNP assembly.

With the autophagy activating kinase ULK1 we identified an unknown player in this complex pathway. Although our data demonstrate a new regulatory function of ULK1 independent of autophagy, the latest studies were also able to link the autophagy pathway with UsnRNP biogenesis by providing evidence of an autophagosome mediated Sm protein degradation pathway during the early phase of UsnRNP biosynthesis (51). The authors showed that only the Sm proteins D1, D2, D3, and B (but not Sm proteins E, F and G) are degraded by autophagy to avoid toxic Sm protein aggregation in a scenario of pICln deficiency prohibiting disturbances in UsnRNP biogenesis. Our results now show that the autophagy activating Ser/Thr kinase ULK1 attributes an additional key regulatory function in UsnRNP biogenesis by direct phosphorylation of pICln. Phosphorylation of pICln by ULK1 and not by ULK2 results in an enhancement of UsnRNP biogenesis (Figure 7A, B). Convenient to this, in cell lines deficient for ULK1 and ULK2, the UsnRNP biogenesis is dramatically reduced and could be reestablished by reconstitution of ULK1 only (Figure 7D). If ULK1 dependent phosphorylation of pICln is blocked the Cajal bodies in the nucleus are altered (Figure 6A–D). The observations from Prusty and colleagues (51) together with our results demonstrate that not only the amount of free available pICln but also the phosphorylation status of pICln is a critical parameter for the storage pool of Sm proteins and an efficient assembly reaction. It will be right of interesting to investigate in the future eg. by using ULK1 deficient cells lacking pICln phosphorylation, how the Sm protein balance is regulated, but even more of interest to answer the question of how the activity of ULK1 is regulated in this pathway. In this context, it is very much relevant to note, that recent studies already demonstrated a connection of the intracellular energy sensor mTOR to the spliceosomal proteins, especially to SmE (52) and to motor neuron development in context to SMA (53). Conversely, work by Cheng (54) and colleagues have shown that overexpression of U1snRNA leads to induction of autophagy. This suggests a cross-link of both pathways and it remains exciting to investigate how this occurs in detail.

The data presented in this work provide the molecular basis of how the transient opening of the 6S ring, catalyzed by ULK1, lowers the energy barrier during UsnRNP biogenesis. The provided data prove the contact area of pICln and SmG as the predetermined breaking point of the 6S ring to initiate the transfer of the open 6S entity onto the SMN complex *in vivo* and show that ULK1 mediated phosphorylation is a crucial regulatory and essential step of efficient UsnRNP biogenesis.

Our data also show that the PRMT5–ULK1-complex conjoins two distinct post-translational mechanisms of regulation in one complex: symmetrical dimethylation and phosphorylation of the 6S complex to allow for the efficient and highly ordered assembly of UsnRNPs.

Core assembly of UsnRNPs and splicing are ubiquitous processes to regulate translation and differential gene expression in eukaryotic cells. A highly ordered and efficient assembly reaction is a prerequisite to keep the responsive gene to protein balance by mRNA transcription of a cell. Mutations or metabolic disorders within this spliceosomal process lead to a dramatic medical outcome like in amyotrophic lateral sclerosis (ALS), *Retinitis pigmentosa*, or spinal muscular atrophy (SMA) in humans. The evolutionary conserved Unc-51-like kinase (ULK1) was first identified in *C. elegans* as the main factor in early neuronal differentiation and axonal elongation (55). The new mechanism of phosphorylation of pICln by ULK1 may help to explain and address specific neuronal aspects associated with inefficient or reduced UsnRNP assembly in this kind of neuronal human diseases. Intensive work will be necessary to understand in more detail the molecular impact of ULK1 within neuronal disorders and the regulation of ULK1activity in this context.

## DATA AVAILABILITY

All data and constructs are available upon request to christoph.peter@uni-duesseldorf.de.

## Supplementary Material

gkab452_Supplemental_File
