# Supplementary material for: An essential role of the autophagy activating kinase ULK1 in snRNP biogenesis
Source: Nucleic Acids Res. 2021 Jun 7;49(11):6437–55. doi: 10.1093/nar/gkab452 (PMC8216288; doi:10.1093/nar/gkab452)
Supplement: gkab452_Supplemental_File [file gkab452_supplemental_file.pdf]

SD Figure 1

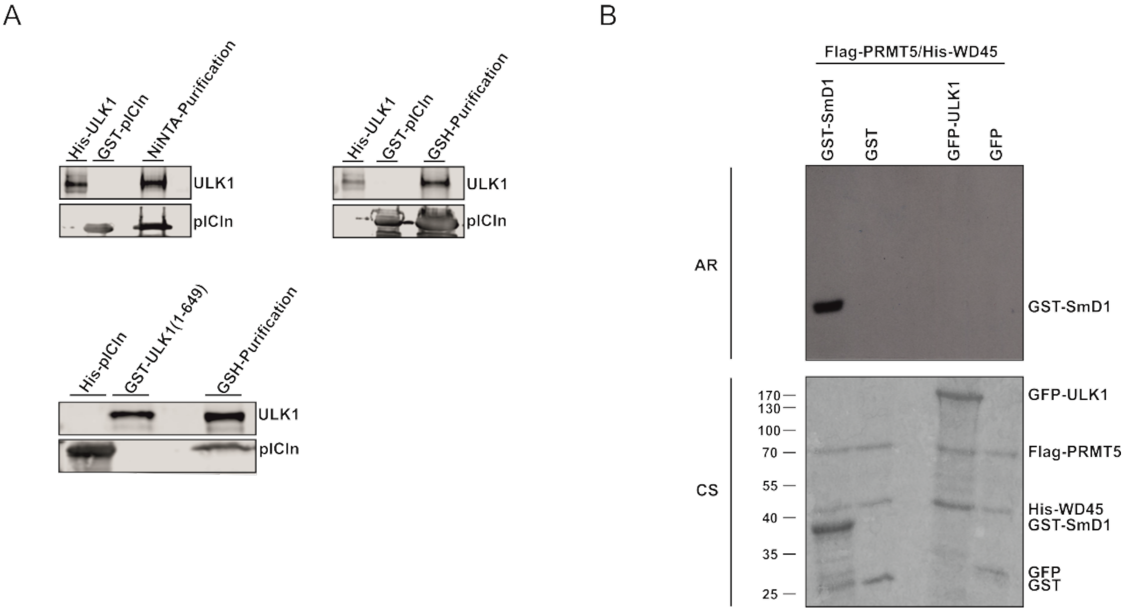

Supplementary Data Figure 1
rULK1 interacts directly with rpICln *in vitro*
A, Recombinant His-ULK1, GST-pICln, GST-ULK1(1-649) or His-pICln were purified by GSH-beads or NiNTA, respectively. Purified rULK1 and rpICln were incubated together for 1,5 h at 4 °C. Subsequently they were re-purified by GSH-beads or NiNTA for 1 h at 4 °C. Respective protein co-binding was analyzed by Tris/Glycine-SDS-PAGE and western blotting using antibodies against ULK1 and pICln. B**,** 1 µg recombinant active Flag-PRMT5/His-WD45 was incubated with GFP-ULK1 or GST-SmD1 (positive control) and 1 µCi [^{3}H]-SAM for 1 h at 37 °C. Samples were separated by Tris/Glycine-SDS-PAGE and analyzed by autoradiography. AR: autoradiography, CS: coomassie blue staining.

SD Figure 2

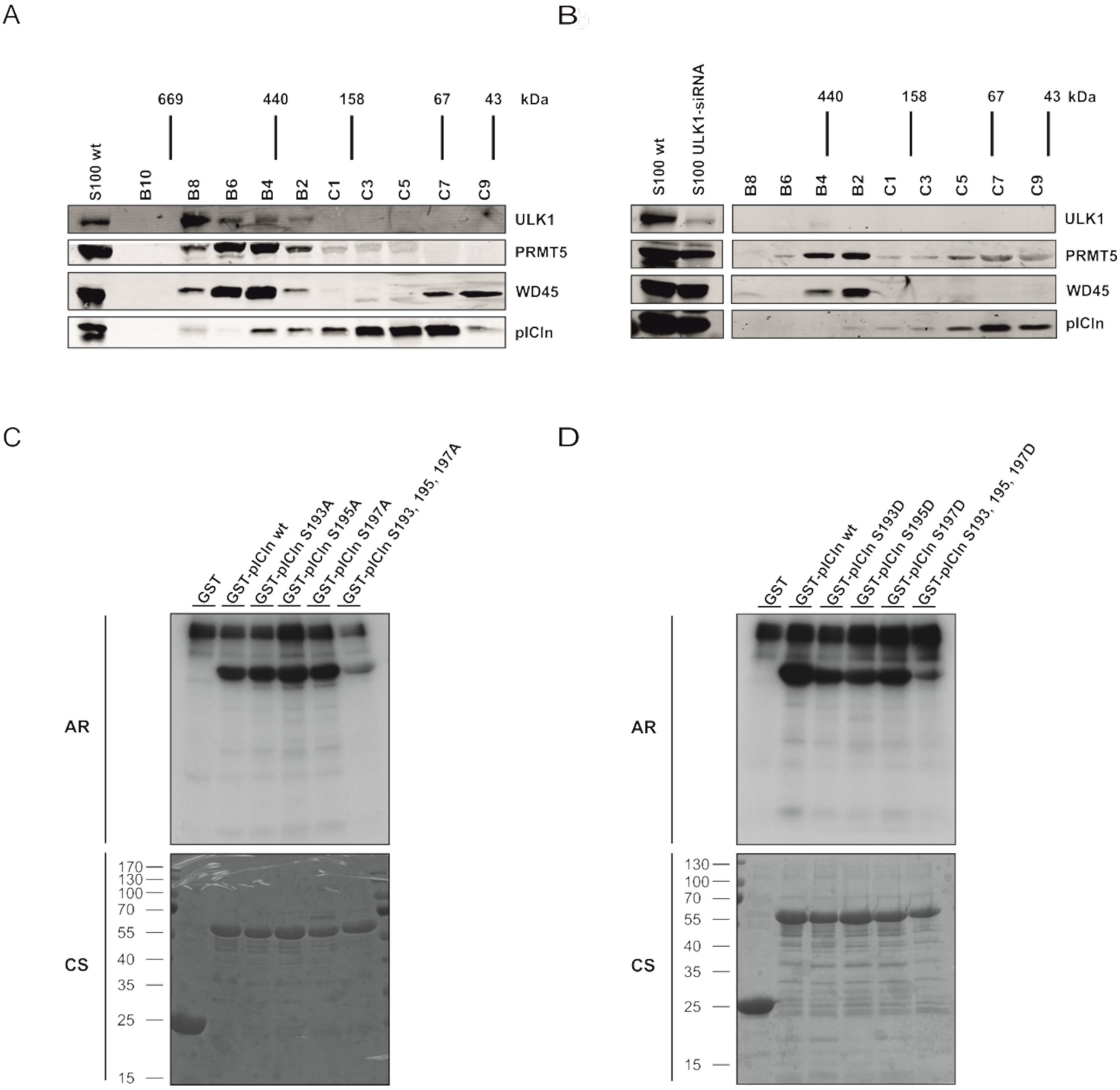

Supplementary Data Figure 2

Western Blot of size exclusion chromatography of wild type HEK293T and HEK293T ULK1-siRNA knockdown S100 extracts

A; B, S100 extract of wild type HEK293T cells (wt) and HEK293T-ULK1-siRNA knockdown (ULK1-KD) cells was resolved by gel filtration, using a Superdex 200 column. Complexes were analyzed by Tris/Glycine-SDS-PAGE and western blotting using antibodies against ULK1, PRMT5, WD45 and pICln. C; D, ULK1 phosphorylates pICln in the C-terminal region on residues S193, S195, and S197. C, *In vitro* kinase assays using recombinant active GST-ULK1 expressed in Sf9 insect cells and GST-pICln wt, -pICln S193A, -pICln S195A, -pICln S197A, -pICln S193, 195, 197A and GST purified from *E. coli* as substrate proteins were incubated with 10 µCi [32P]-ATP for 45 min. at 30 °C. Samples were separated by Tris/Glycine-SDS-PAGE and analyzed by autoradiography. D, *In vitro* kinase assays with GST-pICln wt, -pICln S193D, -pICln S195D, -pICln S197D, -pICln S193, 195, 197D and GST, purified from *E. coli* as substrate proteins, were performed as described in C.

SD Figure 3

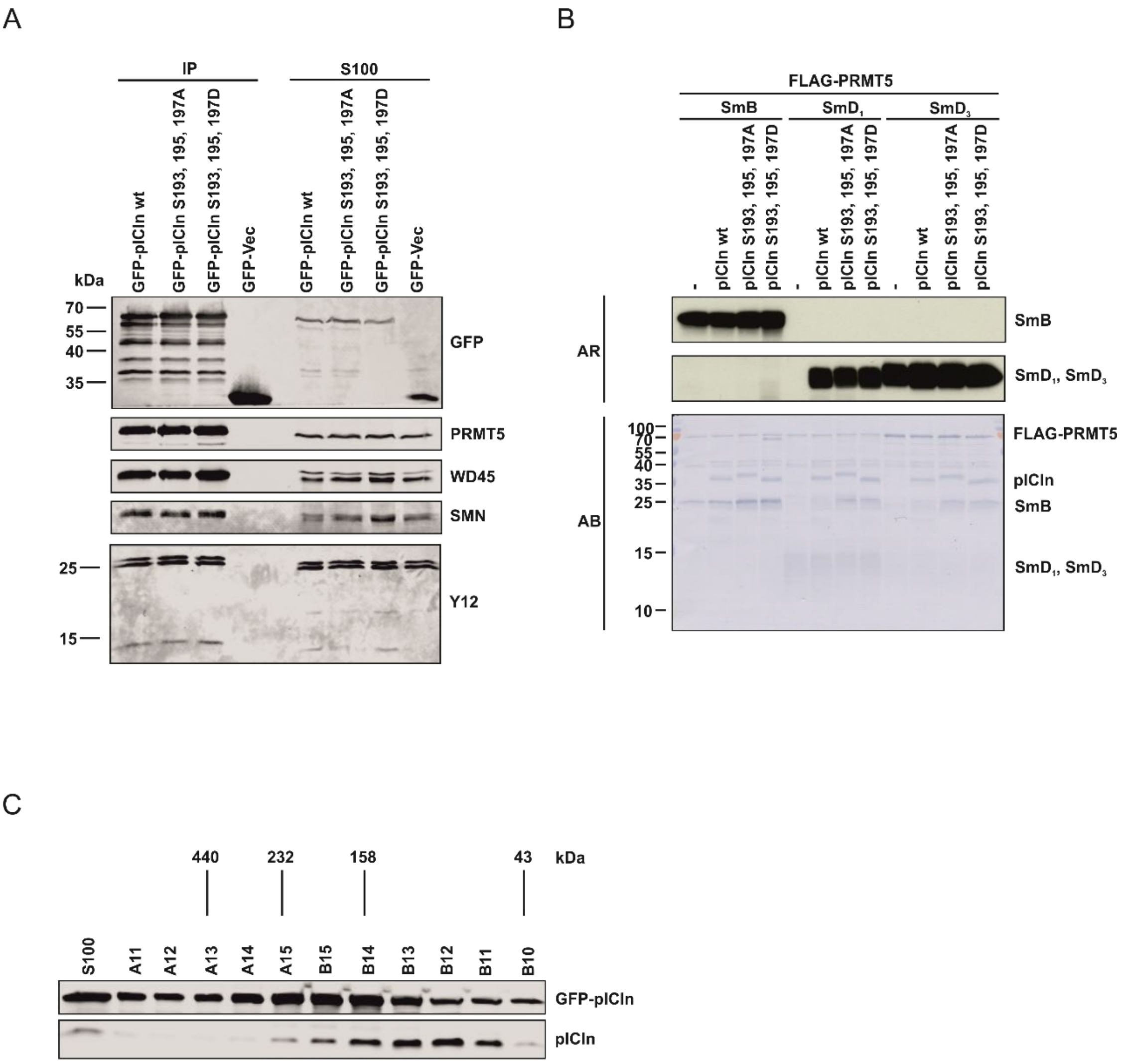

Supplementary Data Figure 3

Composition and activity of the PRMT5-complex with pICln phosphomutants in reference to pICln wt

A**,** GFP-IP was performed and analyzed by Tris/Glycine-SDS-PAGE and western blotting using antibodies against GFP, PRMT5, WD45, SMN and Y12. B**,** Radioactive methylation assay of SmB, SmD1 and SmD3 by PRMT5. 500 ng of Sm substrate proteins were pre-incubated with or without (-) pICln and the corresponding mutants for 30 min. Methylation assay was performed for 1.5 h at 37 °C with 150 ng of active PRMT5. C**,** For velocity analysis, gel filtration was performed with S100 extract of Flp-In T-REx 293-GFP-pICln cells, using a Superdex 200 increase column. Separation of GFP-pICln and endogenous pICln were analyzed by Tris/Glycine-SDS-PAGE and western blotting using antibodies against GFP and pICln.

SD Figure 4

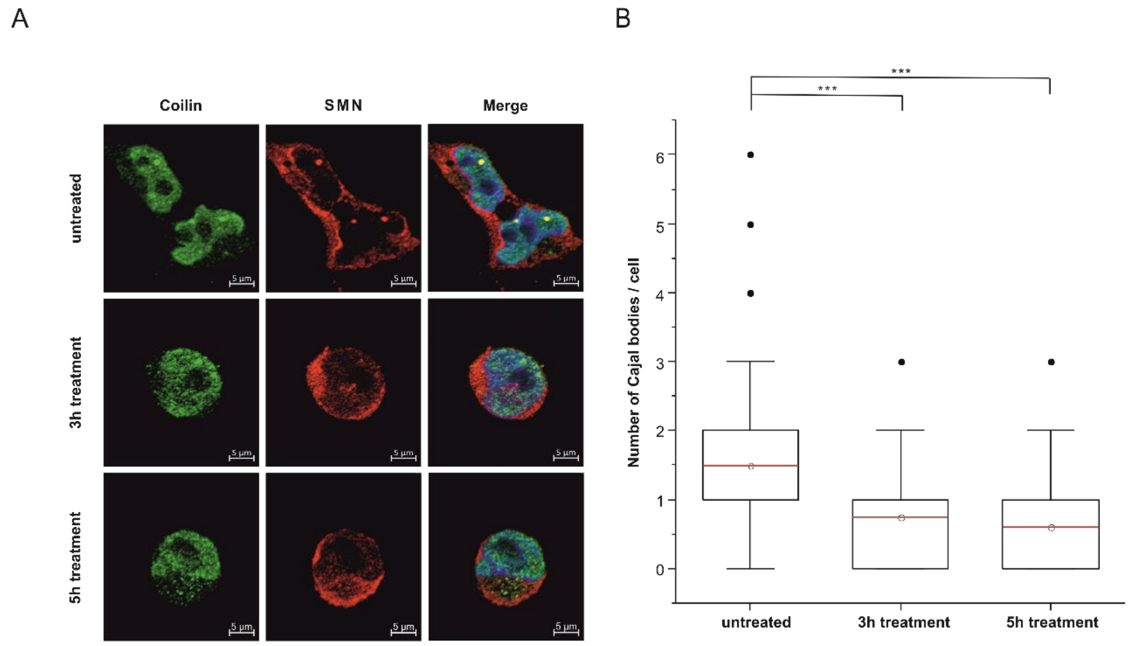

Supplementary Data Figure 4

Inhibition of ULK1 results in a decreased number of Cajal bodies

A; B**,** HEK293T cells were treated with 30 µM ULK inhibitor MRT67307 for 3 and 5 h. A, The cells were fixed and Cajal bodies were visualized with antibody staining against Coilin (green) and SMN (red). DNA was stained with DAPI (blue). B, Inhibition of ULK causes a reduction in the snRNP storage pool. In the boxplot diagram the "box" represents 25-75% of all values and the mean (red), standard deviation and out layers are visualized. HEK293T cells show an average of 1.49 (n = 518) Cajal bodies. Treatment of cells with ULK inhibitor caused a significant decrease in the number of Cajal bodies. The p-value was calculated with Origin using the Mann-Whitney U test. ***$P < 0.005$; scale bars: 5 µm (A)**.**

SD Figure 5

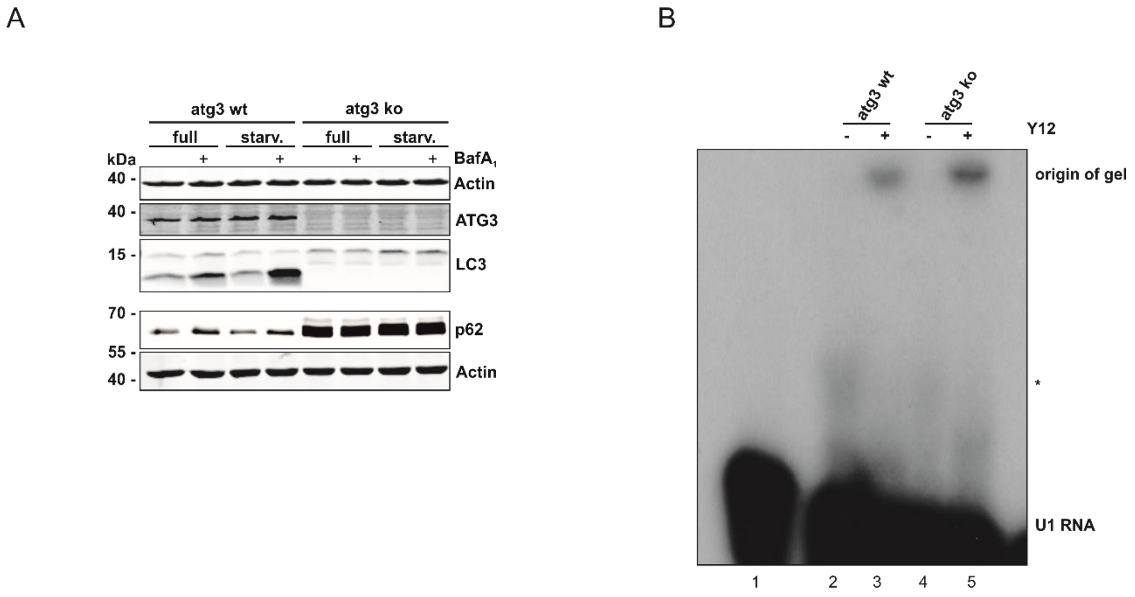

Supplementary Data Figure 5

ATG3 deficient cells are capable for UsnRNP biogenesis

A**,** MEF cells, which express wild type atg3 or are deficient for ATG3, were treated with full or starvation medium (EBSS) in the absence or presence of bafilomycin A1 (BafA1; 10 nM) for 2 h. Afterwards, cells were harvested, lysed and cleared cellular lysates were subjected to SDS-PAGE and immunoblotting for ATG3, p62, LC3, and Actin. B**,** *In vitro* transcribed U1 snRNA labelled with 10 µCi [32P]-UTP was incubated with S100 extracts from MEF cells either lacking ATG3 or cells reconstituted with ATG3 wt. After incubation samples were directly analyzed by native gel electrophoresis or the same samples were subjected to supershift analysis with the Y12 antibody to show the specific formation of snRNPs.

SD Figure 6

```
ICLN_HUMAN MSFLKSFPPPGPAEGLLRQQPDTEAVLNGKGLGTGTLYIAESRLSWLDGS 50
ICLN_DROME MVLIMRVSPP--EHGLLYTANNIKLKLGDKVVGEGTVYIAQNTLSWQPTE 48

ICLN_HUMAN -GLGFSLEYPTISLHALSRDRSDCL--------------GEHLYVMVNAK 85
ICLN_DROME LAEGISIEWKQVSLHGISSNPRKCIYFMLDHKVEWNGVYGDPPQQAVNGR 98

ICLN_HUMAN FEEESKEPVAD---EEEEDSDDDVEP--------ITEFRFVPSDKSALEA 124
ICLN_DROME NGGGSEAEVDEGNGSDEHDEDDNFEDAVDEQFGEVTECWLMPEDIHTVDT 148

ICLN_HUMAN MFTAMCECQALHPDPEDEDSDDYDGEEYDVEAHEQGQGDIPTFYTYEEGL 174
ICLN_DROME MYSAMTTCQALHPDSANSDSEDSDPMQDAGGLEDEAMEEDDALTLGRNGV 198

ICLN_HUMAN SHLTAEGQATLERLEGMLSQSVSSQYNMAGVRTEDSIRDYEDGMEVDTTP 224
ICLN_DROME QNLSLDDDE--ERFEDADE------------------------------- 215

ICLN_HUMAN TVAGQFEDADVDH 237
ICLN DROME ------------- 215
```

Supplementary Data Figure 6
Sequence alignment of human pICln
Sequence alignment (multalin) of human pICln (P54105-1) and drosophila pICln (A1ZAW5-1). Black shade indicates identical amino acids whereas grey shade indicates similar amino acids. Sequence identity: 22.05%. Underlined serines indicate phosphorylation sites of ULK1 in human pICln at positions 193, 195 and 197. These phosphorylation sites are missing in drosophila pICln.
